# Fast Curing Multifunctional Tissue Adhesives of Sericin-Based Polyurethane-Acrylates for Sternal Closure

**DOI:** 10.1021/acsami.2c14078

**Published:** 2022-09-06

**Authors:** Sevgi Balcioglu, Samir Abbas Ali Noma, Ahmet Ulu, Merve Goksin Karaaslan-Tunc, Onural Ozhan, Suleyman Koytepe, Hakan Parlakpinar, Nigar Vardi, Mehmet Cengiz Colak, Burhan Ates

**Affiliations:** † Department of Medicinal Laboratory, Sakarya University of Applied Sciences, 54000 Sakarya, Turkey; ∇ Faculty of Arts and Sciences, Department of Chemistry, Bursa Uludaǧ University, 16059 Bursa, Turkey; ‡ Faculty of Arts and Sciences, Department of Chemistry, İnönü University, 44210 Malatya, Turkey; § Taşkent Vocational School, Selçuk University, 42000 Konya, Turkey; ∥ Medical Faculty, Department of Medicinal Pharmacology, İnönü University, 44210 Malatya, Turkey; ⊥ Medical Faculty, Department of Histology and Embryology, İnönü University, 44210 Malatya, Turkey; # Medical Faculty, Department of Cardiovascular Surgery, İnönü University, 44210 Malatya, Turkey

**Keywords:** bioadhesives, UV-curable, polyurethane-acrylate, sternal closure, sericin

## Abstract

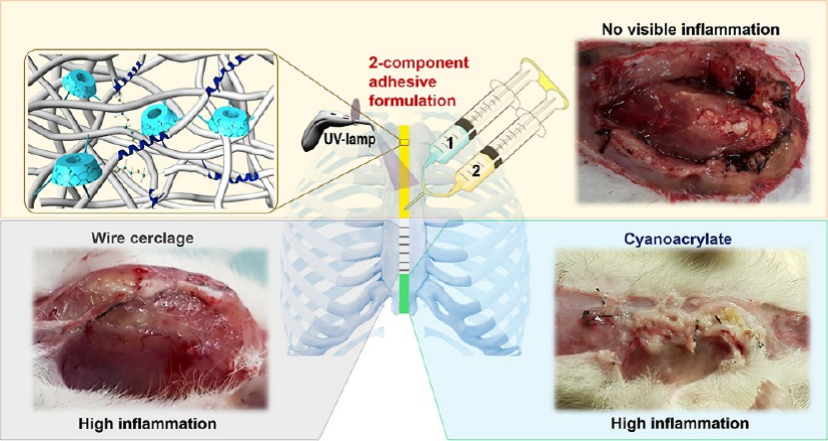

The use of wire cerclage after sternal closure is the standard method because of its rigidity and strength. Despite this, they have many disadvantages such as tissue trauma, operator-induced failures, and the risk of infection. To avoid complications during sternotomy and promote tissue regeneration, tissue adhesives should be used in post-surgical treatment. Here, we report a highly biocompatible, biomimetic, biodegradable, antibacterial, and UV-curable polyurethane-acrylate (PU-A) tissue adhesive for sternal closure as a supportive to wire cerclage. In the study, PU-As were synthesized with variable biocompatible monomers, such as silk sericin, polyethylene glycol, dopamine, and an aliphatic isocyanate 4,4′-methylenebis(cyclohexyl isocyanate). The highest adhesion strength was found to be 4322 kPa, and the *ex vivo* compressive test result was determined as 715 kPa. The adhesive was determined to be highly biocompatible (on L-929 cells), biodegradable, and antibacterial (on *Escherichia coli*, *Pseudomonas aeruginosa,* and *Staphylococcus aureus* bacteria). Finally, after opening the sternum of rats, the adhesive was applied to bond the bones and cured with UV for 5 min. According to the results, there was no visible inflammation in the adhesive groups, while some animals had high inflammation in the cyanoacrylate and wire cerclage groups. These results indicate that the adhesive may be suitable for sternal fixation by preventing the disadvantages of the steel wires and promoting tissue healing.

## 1 Introduction

According to Adult Cardiac Surgery Database (ACSD) 2018 data, worldwide, 6.8 million people annually undergo open-heart surgery, which involves opening the sternum (rib cage).^1,2^ Sternal closure is the longitudinal division of the sternum into two equal parts, especially at the beginning of all open-heart and lung surgeries, to provide access to organs.^3,4^ After the operation, the sternum must be closed again for proper healing, which is achieved by the use of various mechanical sternal fixation materials. Mechanical fixation can sometimes cause greater and more catastrophic problems than the surgery itself. The use of wire cerclage after sternal closure (sternotomy) is still a standard method. In this method, bent stainless steels are placed around the bones.^5^ However, the patient’s age and the experience of the operating surgeon are critical in placing the wires, because, especially in the elderly and osteoporotic bones, steel wires can cut the bone. The morbidity rate here is around 0.5–8%. The surgical procedure is performed under general anesthesia. If the bones rupture, the patient feels intense pain after the effect of postoperative anesthesia wears off. Moreover, this rupture can result in death. This mortality rate is between 10 and 40%.^6,7^ Deaths from bone ruptures are estimated to be as high as 68,000. In the literature, there are also some alternative agents to steel wires such as FiberTape suture. This new cerclage system is nonmetallic, is made of ultra-high-molecular-weight polyethylene, and reduces the risk of bone cut-through.^8^ However, there are no clinical trials in sternal closure yet, and it is not widely used. Therefore, there is a need for new and noninvasive methods such as adhesive application to reduce mortality. However, in emergencies, the sternum may need to be reopened within the first 24 h. Steel wires are suitable agents for reopening.^9^ Therefore, adhesives developed should be designed considering the early reopening of the sternum.^3^

The main purposes of bone and tissue bonding are to prevent biological fluid leakage and minimize trauma. Although mechanical methods are widely used in these procedures, they have many disadvantages such as tissue trauma, operator-induced failures, and the risk of infection. Because of these disadvantages, it is essential to develop surgical adhesives. Surgical adhesives constitute an alternative to mechanical methods due to their ease of use, reduction in operation time, impermeability of body fluids, biodegradability, and noninvasive nature.^10^ Therefore, the use of tissue adhesives in surgical operations has gained great importance recently, and they were used in approximately 35.2% of surgical operations in 2010.^11^ Furthermore, adhesives have been tried as an alternative and supportive to steel wires in sternotomy, and positive results have been obtained. Hashim et al. used Kryptonite, a polymer-based bone cement, in the sternal closure process and found that it reduced the level of pain compared to steel wires by measuring serum cytokines, which is a pain marker. Therefore, they gave less analgesic to the adhesive-applied group than the steel wire group. They also reported that in cases requiring reopening, the glued sternum was opened easily after 24 and 48 h.^12^ Fedak et al. found that the Kryptonite-enhanced sternal closure was safe and supportive to conventional closure with stainless steel wire, with proven benefits on functional recovery, respiratory capacity, incisional pain, and analgesic requirements.^13^ Bayramoglu et al. reported that Kryptonite bone cement, when combined with a standard wire cerclage, increases mechanical strength, prevents sternal dehiscence, reduces postoperative pain, and improves the quality of life after conventional cardiac surgery.^14^ In similar studies with Kryptonite, great advantages were also observed compared to steel wires, and it was determined that it prevents major sternal complications.^1,13−16^ Kryptonite is a castor oil-based and US Food and Drug Administration (FDA)-approved polymeric bone cement. However, it is no longer available on the surgical market. Nevertheless, it promises that tissue adhesives can be used in sternal closure due to the positive results obtained in the studies. Zhang et al. used 2-octyl cyanoacrylate for the adhesion of the upper skin after sternotomy and found that the level of infection was reduced.^17^ A similar study was done by Chambers and Scarci, and similar results were found.^18^ Furthermore, quick adhesion in adhesives can be inconvenient if the adhesive is not elastic (cyanoacrylates). In reopening situations, it is difficult to remove these adhesives without damaging the tissue. Therefore, there is a need to develop more flexible adhesives to remove them easily from the surface without damaging tissue. According to a study by Sidhu et al., when the adhesive-free steel wires are removed, they can lead to deep sternal wound infection (DSWI) in reopen-requiring cases. Moreover, they found that in the adhesive groups, the infection was reduced by preventing bacterial adhesion as the adhesive infused to the leaks.^19^ Watanabe et al. in 1997 used microfibrillar collagen hemostat powder and antibiotic-containing fibrin glue to prevent sternal bleeding and achieved successful results.^20^ In the sternotomy performed on the cadaver, Mehrvar et al. compared glass polyalkenoate cement with steel wires and found that it prevented DSWI.^21^ In the literature, adhesive trials are being conducted to eliminate the disadvantages of sternotomy with steel wires, especially DSWI. However, most of these formulations are not exactly an adhesive and aim to create a filler in the sternal region and thus prevent sternal bleeding and DSWI. Furthermore, none of these formulations has been tested in an *in vivo* rat model. For this reason, in our study, UV-curable, polyurethane-acrylate (PU-A) adhesive was synthesized and applied in the sternotomy model designed on rats.

**Figure 1:**
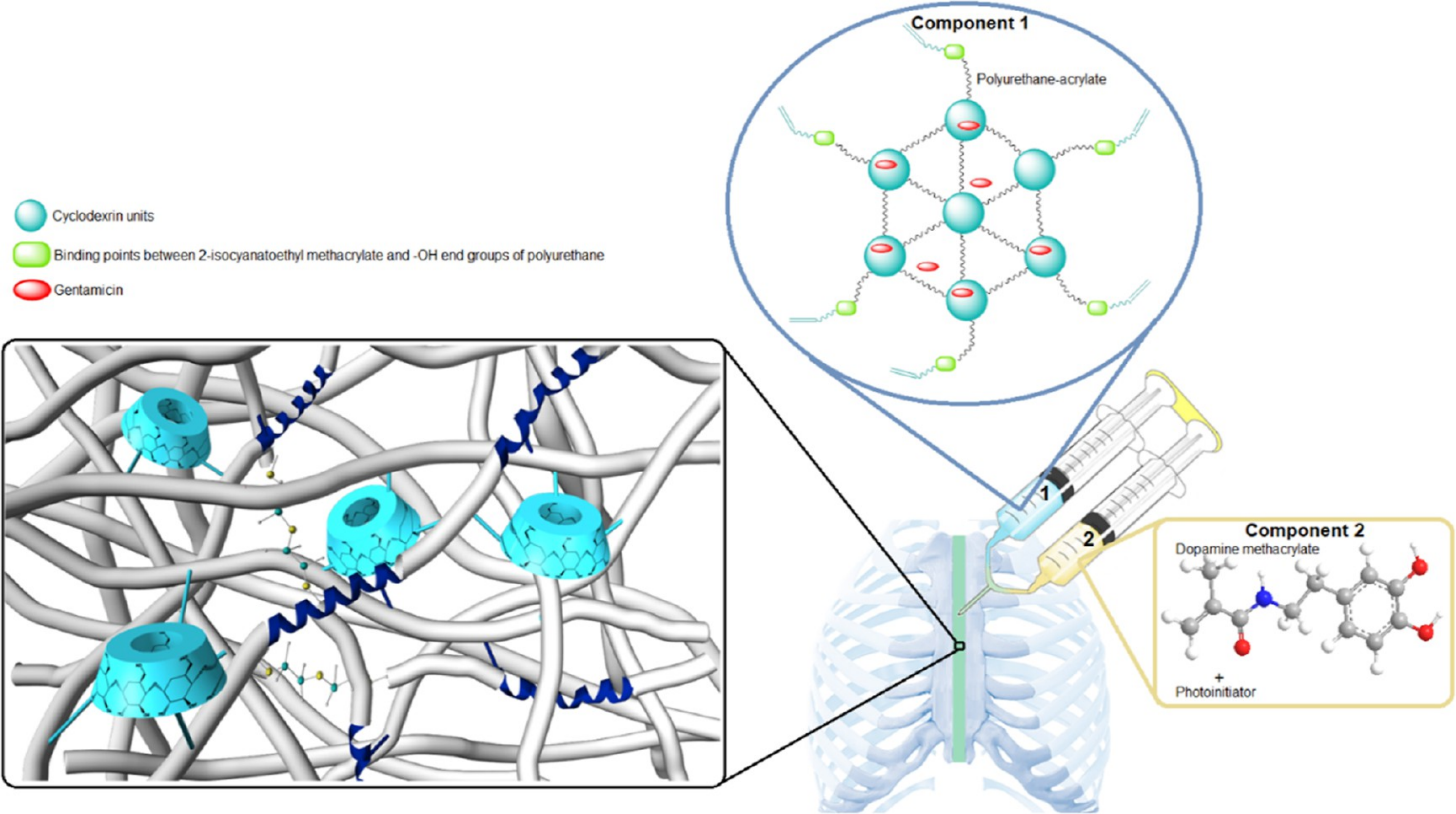
Sternal adhesive application diagram.

The reason is for choosing PU-A as the adhesive in our study, having a hyperbranched 3D structure, spreadable consistency, and UV-curable structure. In addition to these, PU-As are frequently preferred in different fields such as coating materials, nanocomposites, adhesives, and membranes with their high biocompatibility properties, high adhesion strength, high solubility properties, and different cross-linking mechanisms.^22,23^ It is thought that the high thermal stability and mechanical strength of the PU-A structure will also prevent the wrong opening of the sternum by providing mechanical support in intrathoracic pressure. Polymethyl methacrylate (PMMA) bone cements are also used in orthopedic fixation devices due to their high thermal stability and mechanical strength. However, PMMA is fragile and does not adhere to bones.^24^ For this reason, the PU-A structure, which has more flexibility and high adhesion strength, has a critical place in sternum fixation. PU-A synthesis was carried out in two steps in our study. In the first step, the PU structure was synthesized using different polyols, cross-linkers, and isocyanate, and then, the structure was double bond-functionalized with 2-isocyanoethyl methacrylate. Thus, the polymer has gained photoreactive (UV-curable) feature, which provides fast curing property to the polymer (Figure 1). This feature also enables the polymer to harden in the tissues without heating. PMMA bone cement and cyanoacrylates are composed of fast-curing pre-polymers, but they heat during curing and damage tissues.^25,26^ The main cross-linking (curing) in PU-A is provided by UV light. However, in addition, another cross-linking mechanism is envisaged with mussel-mimetic regions in humid environments. Sea mussels can adhere tightly to almost all surfaces (rock, mica TiO_2_, polystyrene) and even adhesion-resistant PTFE surfaces in wet and irregular environments.^27,28^ This adhesion ability includes perceiving the selected surface thanks to the foot of the mussels followed by the adhesion process through released proteins [mussel foot proteins (mfp)] from this area. mfp proteins contain different ratios of 3,4-dihydroxyphenyl-l-alanine (DOPA) formed by post-translational modification of tyrosine.^29−31^ DOPA provides adhesion in wet environments due to the hydrogen bond, metal–catechol coordination, electrostatic interactions, and cation−π and π–π aromatic interactions.^27,32,33^ This functionality in our adhesive is provided by dopamine methacrylamide containing catechol groups.

In post-operative sternum surgery, intensive antibiotic treatment is applied in case the patient is infected. Especially Vancomycin and Gentamicin drugs are used for these treatments.^34^ Therefore, Gentamicin was added to the adhesives for topical antibiotic release to prevent possible infections. Here, the inclusion complex property of β-cyclodextrin, which is also used as a cross-linker, with Gentamicin has been utilized.^35^ Due to the 21 −OH it contains, β-cyclodextrin contributes to the cross-linking of polymers and has high biocompatibility.^36^ Additionally, the sericin protein is covalently attached to the structure to increase the biocompatibility and biodegradability of the adhesive and to provide extra adhesiveness. Sericin is one of the two proteins in silk structure, and it wraps the other protein, fibroin fibers, with an adhesive layer. Sericin consists of 18 different amino acids, 32% of which is serine. In this way, it contributes to adhesion by making hydrogen bonds with different biological surfaces. It is also easy to work with its soluble feature and has pharmacological, cosmetic, and biotechnological applications.^37,38^

Lastly, flexibility was regulated by adding different sizes of polyethylene glycols (PEG) to the structure so that the adhesive could withstand the thoracic internal pressure. PEGs and aliphatic isocyanate used in the structure have contributed to high biocompatibility and biodegradability as well as flexibility. Thus, a 2-component adhesive system was developed in our study, and it was successfully applied in the *in vivo* sternal fixation model in rats (Figure 1). Component 1 contains the synthesized PU-A structure, while component 2 contains the photoinitiator and dopamine methacrylamide.

## 2 Experimental Section

### 2.1 Instruments and Materials

Chemical and physical characterizations were performed using Shimadzu TGA-50, Shimadzu DTA-50, Shimadzu DSC-60, Park Systems XE-100E AFM, LEO-EVO 40× SEM, Bruker NMR 300 MHz Ultrashield, Ascend 600 ULW, and PerkinElmer UATR Two FTIR devices. All spectrophotometric analyses were carried out with a Shimadzu UV-1601 UV–visible spectrophotometer and BioTek Eon Elisa microplate reader. The adhesion results were obtained with an MTS E42 test analyzer. L-929 cell morphologies were determined by a JuliFL cell analyzer and an Olympus CKX41 inverted/fluorescence microscope. Histological analysis was carried out with Leica DFC-280 microscope and Leica Q Win Image Analyze System (Leica Micros Imaging Solutions Ltd., Cambridge, UK).

Polyethylene glycol 200 (PEG200), polyethylene glycol 400 (PEG400), and polyethylene glycol 600 (PEG600) were purchased from Merck. 4,4′-Methylenebis cyclohexyl diisocyanate, β-cyclodextrin, silk sericin (10–40 kDa), toluene-4-sulfonyl isocyanate, tetrabutylammonium hydroxide, MTT (thiazolyl blue tetrazolium bromide), Dulbecco’s modified Eagle’s medium (DMEM), gentamicin sulfate, and 2-isocyanatoethyl methacrylate were purchased from Sigma-Aldrich. DMSO, acetonitrile, methanol, ethanol, and other solvents were obtained from Merck. Irgacure-2959 was purchased from TCI (Tokyo Chemical Industry). All chemicals were used without further purification.

### 2.2 Synthesis of Pre-polymers

Polymer synthesis was carried out in two stages. PU synthesis was carried out in the first stage, and PU-A (pre-polymer) synthesis was carried out in the second stage. The NCO/OH ratio for PU synthesis was theoretically set to 1/1.2, 1/1.3, and 1/1.4. 4,4′-Methylenebis cyclohexyl diisocyanate (HMDI) as the NCO agent and PEG200/PEG400/PEG600, β-cyclodextrin (β-CYC), and silk sericin as OH agents were used. Increasing the ratio of OH was achieved by increasing the ratio of PEG by keeping constant the ratio of other agents. Experimental OH levels were measured according to the method given under “Determination of Hydroxyl Ratios in the Polymers”. The reaction was carried out under reflux and inert atmosphere at 75 °C for 24 h with DMSO as a solvent. The reaction was terminated with the complete disappearance of the free isocyanate peak at 2265 cm^–1^ by Fourier transform infrared (FTIR) spectroscopy.^39^ The nomenclature of the PU samples and the stoichiometric ratios of the monomers are shown in Table 1. Stoichiometric ratios were determined according to the number of −OH and −NCO. HMDI contains 2 −NCO ends, while PEGs (no matter the molecular weight) contain 2 −OH ends. Thus, for example, in the synthesis of HMDI-SER-P200-20 PU, 1 mol of PEG, 0.1 mol of sericin, and 0.1 mol of β-CYC were added to 1 mol of HMDI.

**Table 1:** PU Nomenclatures and Stoichiometric Ratios of the Monomers

sample name	PEG200	PEG400	PEG600	β-CYC	sericin	HMDI
HMDI-SER-P200-20	105			10	5	100
HMDI-SER-P200-30	115			10	5	100
HMDI-SER-P200-40	125			10	5	100
HMDI-SER-P400-20		105		10	5	100
HMDI-SER-P400-30		115		10	5	100
HMDI-SER-P400-40		125		10	5	100
HMDI-SER-P600-20			105	10	5	100
HMDI-SER-P600-30			115	10	5	100
HMDI-SER-P600-40			125	10	5	100

In the second stage of the synthesis, after determining the amount of OH in the PU structures, 2-isocyanatoethyl methacrylate was added in a ratio of 1/1 relative to OH under inert argon atmosphere for 5 h and the pre-polymer structures were obtained. The resulting polymers were named pre-polymer and coded as ...-A (e.g. HMDI-SER-P200-20-A).

### 2.3 Determination of Hydroxyl Ratios in the Polymers

ASTM E1899-08 potentiometric titration standard method was used to determine the free hydroxyl (−OH) groups in PU structures.^40^ In this method, potassium hydrogen phthalate (KHP) was used to determine the correlation factor (*f*) of the standard. KHP was dried at 120 °C for 2 h and then kept in a desiccator for 1 h. 180 mg of KHP was dissolved in 60 mL of pure water, and 5 mL of toluene-4-sulfonyl isocyanate (TSI) prepared in acetonitrile was added. Then, the mixture was titrated with 0.1 M tetrabutylammonium hydroxide (TBAOH) prepared in methanol to the turning point.
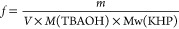
where *f* is the correlation factor, *m* is the standard amount (mg), *M*(TBAOH) is the molarity of TBAOH (mol/L), Mw(KHP) is the molecular weight of KHP, and *V* is the titrant volume spent for the first turning point (mL).

After this step, to determine the hydroxyl number of the samples, 100–500 mg of PU samples were mixed until dissolved in 5 mL of acetonitrile. Then, the reaction was started by adding 5 mL of TSI, and the mixture was closed with parafilm to prevent it from being affected by the humidity of the air. After 10 min, 0.25 mL distilled water was added and blended for 5 min to blind unreacted isocyanates. Then, 10 mL acetonitrile was added to the mixture and titrated with 0.1 M TBAOH until the second turning point. OH numbers were calculated for each formulation by using the formula below.
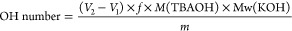
where *V*_2_ is the second turning point (mL), *V*_1_ is the first turning point (mL), *f* is the correlation factor, *M*(TBAOH) is the molarity of TBAOH (mol/L), Mw(KOH) is the molecular weight of KHP (g/mol), and *m* is the sample amount (g).

The data obtained as a result of the experiments were plotted as % change in potential versus titrant volume. As a result of the calculations, the free OH value in PUs was given as mg potassium hydroxide (KOH) per g sample.

### 2.4 Gentamicin Immobilization to the Pre-polymers

Gentamicin, the most commonly used systemic antibiotic after sternal operations, was used to provide antibacterial property to the pre-polymers. The amount of Gentamicin was adjusted to be 2.5% by weight. This concentration was determined from the literature as the most effective dose in the study on bone cements.^41^ Gentamicin was added to the polymer solution and mixed for 2 h at room temperature to homogeneously disperse and complete the guest–host interaction with β-cyclodextrin. Their solvents were removed under vacuum.

### 2.5 UV-Curing Processes of Pre-polymers

For curing of the pre-polymer structures, the samples were exposed to 365 nm UV light for 5 min with photoinitiator and dopamine methacrylamide to fully solidify the pre-polymers. In the study, it was determined that the synthesized pre-polymer structures were cured in 30 s. However, depending on the amount of glue applied, it takes time for the light to penetrate deeper. For this reason, the polymers were cured for 5 min to complete the reaction, to reach the maximum adhesion strength, and to disappear the monomers completely as the unreacted double bond is toxic. 1% of Irgacure-2959 (2-hydroxy-4′-(2-hydroxyethoxy)-2-methylpropiophenone) was used as photoinitiator.^42^ First, to provide spreadability, 1 g of pre-polymer was weighed and dissolved in 500 μL of ethanol (99.9%). In another tube, 10 mg of Irgacure-2959 and 10 mg of dopamine methacrylamide were dissolved in 50 μL of ethanol and added to the pre-polymer solution. Then, the pre-polymer-Irgacure-2959-dopamine methacrylamide mixture was cured for 5 min under UV light. There were two mechanisms for the curing process. The basic one was opening and cross-linking of the double bonds, whereas the second one was providing mussel-mimetic units. Dopamine methacrylamide was used to provide the mussel-mimetic units, thanks to catechol functional groups. In this way, the samples could be cured faster with two different mechanisms. The resulting product is a highly cross-linked, transparent polymer in an insoluble solid form. Dopamine methacrylamide was synthesized from dopamine and methacrylate anhydrite according to Glass et al.^43^ The adhesives that were not exposed to UV light were named ...- A while UV-cured forms were named ...- AC.

### 2.6 Structural, Thermal, and Morphological Characterization of Final Adhesives

For synthesized adhesive formulations, FTIR spectroscopy was used to determine the functional groups formed/lost at each stage. In the analysis, the scanning range was determined as 400–4000 cm^–1^. NMR analysis was performed on the synthesized PU and pre-polymers to prove that the structure became double bond functional. Because the final formulations were not in a soluble form, NMR analysis could not be performed on these samples. DMSO-*d*_6_ was used as the solvent for NMR analysis.

Thermogravimetric analysis (TGA), differential scanning calorimeter (DSC), and differential thermal analysis (DTA) techniques were used to determine the thermal behavior and glass transition temperatures (*T*_g_) of the samples before and after UV curing to determine their applicability for sternal closure operations. TGA analysis was performed in the range of 25–600 °C, DTA analysis in the range of 25–500 °C, and DSC analysis in the range of −40–100 °C. Liquid nitrogen was used for DSC cooling processes.

Scanning electron microscopy (SEM) and atomic force microscopy (AFM) analyses were performed on selected samples to determine the general morphological structures of the final polymer formulations after UV curing. In AFM analysis, a needle tip provides high resolution three-dimensional imaging of the surface. The film samples were scanned non-contact with AFM tips, and the topology was extracted. SEM is a testing process that scans a sample with an electron beam to produce a magnified image for analysis and gives insights into the surface topographies of the film samples.

### 2.7 Determination of *In Vitro* Adhesive Strengths of Final Adhesives

Transparent glass slides were used to determine the *in vitro* bond strength of the adhesives. The experiment was carried out in accordance with ASTM (F2255-03, “Test Method for Strength Properties of Tissue Adhesives in Lap-Shear by Tension Loading”) standards.^44^ The lap shear adhesion test was performed by applying 50 mg polymer sample to 2.5 × 1 cm^2^ area with MTS brand Mechanical Test Analyzer. At first, 50 mg of pre-polymer sample was dissolved in 25 μL of ethanol in a tube. This part shown in Figure 1 was called component 1. A mixture of 0.5 mg Irgacure-2959 and 0.5 mg dopamine methacrylamide was prepared in 2.5 μL of ethanol in another tube. This was also called component 2 as seen in Figure 1. This two-component product was mixed on the slide, and the other slide was placed on the specified area and pressed lightly. Then, these slides were left for 5 min under UV light and the adhesion strength was measured after 15 min. Because of the transparent properties of the slides, the pre-polymers were cured quickly. To determine whether dopamine methacrylamide was bond to the structure, Fe^3+^ was added to the polymer and the Fe-dopamine chelate structure was seen from the green color formed. The chemical binding of dopamine methacrylamide to the polymer structure was indicated. The experiments were carried out with at least 3 replicates, and the results were given in kPa.

### 2.8 Determination of *Ex Vivo* Compressive Strengths of Final Adhesives

After the bovine bone was removed from the animal, it was washed with phosphate buffered saline (PBS) to clean it and lightly dried with a paper napkin. It was then used directly in the experiment. To determine the compressive strength of the adhesives, the bone tissue taken from the bovine rib area was cut in half, the adhesives were applied between them, and UV curing with a wavelength of 365 nm and power of 8 W/cm^2^ was performed for 5 min (see the compressive strength results). Then, the glued bone was placed in the MTS brand mechanical test analyzer with the left arm fixed and the right arm free of the device. The apparatus performing the compression test applied pressure on the bone at a speed of 0.02 mm/s from the top and the compressive strength of the adhesives was measured.^45^ The experiments were carried out with at least 3 replicates and the results were given in kPa.

### 2.9 Determination of Biodegradability, Gentamicin-Release, and Antibacterial Properties of Final Adhesives

According to the adhesion strength results, one PU-A was selected to determine the biodegradability. For the test, the UV-cured film samples were cut to be 1 × 1 cm^2^ and left in 5 mL of PBS (pH 7.4) buffer.^46,47^ These samples were incubated at 37 °C for 4 weeks. Samples were taken at 0, 1, 2, 3, and 4th weeks and dried and weighed again. Degradation graphs were drawn from % mass loss. The experiment was carried out with at least 3 replicates.

To examine the gentamicin release kinetics, 0.1 g of samples were prepared and immersed in 10 mL of PBS (pH 7.4, 50 mM) solution. Sample solutions were incubated at 37 °C for 5 days, and 100 μL of the sample was taken at specific time intervals (0.25, 0.5, 1, 2, 4, 24, 48, 96, and 120 h), and fresh PBS was added instead. The solution was filtered through filters with 0.45 μm pore size. The released Gentamicin was derivatized with *o*-phthalaldehyde (OPA). For the derivatization process, 100 μL of OPA solution (5 mg OPA + 100 μL of pure ethanol + 5 μL of β-2-mercaptoethanol + 10 mL PBS) was added to the solution and incubated for 2 min. The resulting complex was measured in a UV–visible spectrophotometer at 340 nm, and the amount of time-dependent Gentamicin was given in %. Gentamicin sulfate solution with a known concentration was used for the standard calibration curve. The experiment was run with at least 3 replicates.^48^

The agar disc diffusion method was used to determine the antibacterial activity of the final adhesive formulation.^49^*Escherichia coli* (ATCC 25922; Gram-negative), *Pseudomonas aeruginosa* (ATCC 27853; Gram-negative), and *Staphylococcus aureus* (ATCC 23235; Gram-positive) were used as microorganisms. Luria-Bertani broth was prepared for *E. coli* and *P. aeruginosa*, and tryptic soy broth was prepared for *S. aureus* for optimum growth. In the preparation of standard Gentamicin discs, Gentamicin was dissolved in distilled water and absorbed into discs in an equivalent (1.25 mg/disc) amount to Gentamicin in the samples followed by sterilizing with UV for 30 min. 50 mg of PU-A adhesive formulations were used. Additionally, Luria-Bertani agar Petri dishes for *E. coli* and *P. aeruginosa* and tryptic soy agar Petri dishes for *S. aureus* were used. Each Petri dish was spread with a spread plate technique of 100 μL from one of three microorganisms corresponding to 0.5 McFarland standard turbidity at 37 °C. In the middle of the Petri dish, a hole equal to the length of a disc was drilled, and the materials were placed there. Additionally, a polymer disc without Gentamicin was placed as a control for each Petri dish. Each Petri dish was studied in duplicate. The Petri dishes incubated at 37 °C for 24 h were observed and photographed, and the antimicrobial effects of the materials were determined by measuring the zone diameters on the agar.

### 2.10 *In Vitro* Biocompatibility Properties

In the biocompatibility test of the final adhesive formulations carried out by the indirect method, cytotoxicity values were determined spectrophotometrically by the MTT (thiazole blue tetrazolium bromide) test.^51^ The experiment protocol was prepared according to ISO-10993-5 “Biological Evaluation of Medical Devices” standards, and *Mus musculus* mouse fibroblasts (L-929) were used in the study. First, the samples were washed with sterile PBS (pH 7.4) and sterilized under UV light for 1 h and then incubated with DMEM medium in an incubator containing 5% CO_2_ at 37 °C for 72 h. The L-929 cell line was proliferated in DMEM for the experiment until it became 80% confluent in the incubator containing 5% CO_2_ at 37 °C, then the cells were removed from flasks with a 0.25% trypsin–EDTA solution. The cells taken by centrifugation at 2000 rpm for 5 min were then added to 96-well plates as 10^4^ cells/well and incubated for 24 h under the same conditions. At the end of the incubation, the medium exposed to the samples was applied to the cells and incubated for an additional 24 h under the same conditions. The medium was then removed from the cells, and 90 μL of fresh medium and 10 μL of MTT solution (5 mg/mL, in PBS) were added to the plates and incubated for 4 h under the same conditions in dark. At the end of the period, the liquid was removed from the cells and exchanged with 100 μL of DMSO. The absorbance of the purple color formed was measured at 550 nm at the Elisa microplate reader. The medium, which was left in the incubator for 72 h, was added to the control wells, and these wells were accepted as 100% alive. % cell viability was calculated and cell morphologies were determined by JuliFL cell analyzer and % confluent ratios were given.

### 2.11 Sternal Closure Model on Rats

For the sternal closure model, an ethics committee report (2016/A-27) was obtained from İnönü University Medical Faculty. Sternal closure was performed with 24 male Wistar rats. In the experiment, while the animals were under anesthesia [ketamine (75 mg/kg) + xylazine (8 mg/kg)], the sternum regions were divided into two parts longitudinally with the help of loop glasses and dental drill, and bleeding control was achieved by cauterization (see the Section 3.8). The head and end parts of the separated sternum were tied with a steel wire and then sealed with the selected formulation. The groups were formed according to Table 2. Adhesion was achieved by exposure to UV curing for 5 min (group 4). In the control group, only the chest area was opened, and the sternum was not opened (group 1). The sternum was opened in the steel wire group, and the closure was carried out with steel wires, the traditional method (group 2). Furthermore, the success of the procedure was followed by comparison with a Venablock embolizing agent (cyanoacrylate), a commercial surgical adhesive (group 3). In this group, 2 steel wires + cyanoacrylate were applied instead of the adhesive. Later, the skin was sutured twice as the upper and lower skin. Rats were sacrificed under anesthesia at the end of 1 week, and surrounding tissues were removed and examined histologically and biochemically.

**Table 2:** Groups of Sternal Closure Model in Rats

group 1	control (*n*: 6)
group 2	steel wire (*n*: 6)
group 3	embolizing agent (cyanoacrylate) (*n*: 6)
group 4	adhesive formulation (*n*: 6)

### 2.12 Histological Evaluation

The sternum removed at the end of the experiment was detected in 10% formaldehyde after the decalcification process. At the end of the determination, the tissues washed in tap water were dehydrated and polished and embedded in paraffin. Sections 4–5 μm thick from paraffin blocks were stained with hematoxylin–eosin (H–E) and Masson trichrome (MT). Histological evaluations were made in terms of inflammatory cell infiltration. Infiltration was scored as 0: no, 1: mild, 2: moderate, and 3: severe in 10 areas randomly selected around the sternum.

### 2.13 Biochemical Evaluation

Inflammation parameters of tissue samples were determined biochemically with myeloperoxidase (MPO)^50^ activity and nitrite oxide (NO) level (Cayman Nitrate/Nitrite Colorimetric Assay Kit).^47^ Serum levels of creatinine (CR) and blood urea nitrogen (BUN) indicating kidney function were determined using the Olympus Autoanalyzer (Olympus Instruments, Tokyo, Japan).

### 2.13.1 MPO Activity

0.1 g of tissue samples was weighed and left in 1 mL of 0.05 M phosphate buffer (pH 6) and homogenized under ice isolation. Then, centrifugation was carried out at 15,000*g* for 15 min at 4 °C. The pellets were separated from the supernatant and added to 500 μL of HETAB solution (0.1% in phosphate buffer). The mixtures were sonificated for 15 s and frozen and thawed again. The thawed samples were likewise sonificated–frozen–thawed and then sonificated for the last time. Afterward, the samples were centrifuged at 15,000*g* for 15 min, and the supernatant was taken. 25 μL of the supernatant and 200 μL of the reaction mixture [28.4 μL of 50% H_2_O_2_ solution and 4.175 mg of *o*-dianicidine in 25 mL of phosphate buffer (pH: 6)] were added to 96 well-plates, and after 5 min, absorbances were measured at 460 nm with a microplate reader. MPO activity results were given as U/g wet tissue.

### 2.13.2 Nitric Oxide Measurement

The nitric oxide level in the tissues was determined using Cayman Nitrite/Nitrate Colorimetric Assay Kit. Tissues were homogenized in PBS (pH: 7.4) (1/5 tissue/PBS ratio). The method was carried out by measuring the azo dyestuff formed at 540 nm resulting from the reaction between Griss dyes and NO_2_. NO results are given as nmol/g wet tissue.

### 2.14 Statistical Analysis

All statistical analyses for *in vitro* studies were performed with a GraphPad Prism 8 program “one-way anova” test, and *p* < 0.05 was considered significant in the results. Data were shown as arithmetic mean ± standard deviation.

Statistical analysis for histological studies was performed with the IBM SPSS statistical software program (SPSS for Windows version 22, SPSS Inc., Chicago, IL). Comparison between groups was made with Anova (Tamhane or Tukey) test for normally distributed data and the Kruskal–Wallis *H* test for non-normally distributed data. Data were expressed as median (minimum–maximum) or arithmetic mean ± standard deviation depending on the distribution. *p* < 0.05 was considered significant.

## 3 Results and Discussion

Synthesis, characterizations, and *in vitro* and *in vivo* biological activities of PU-A polymeric material that can be used in sternum closure were performed. In the structural design of these materials, sericin was used to increase adhesiveness and biocompatibility, cyclodextrin as a cross-linking unit, and PEG as a soft segment. To obtain an injectable formulation, molecular sizes of PEG units were determined as 200, 400, or 600.

In the study, there are three basic cross-linking mechanisms. The first mechanism was use of β-cyclodextrin and sericin as cross-linking agents in PU synthesis. These monomers formed the cross-link points. Additionally, three different molecular weights of PEG (200, 400, and 600) were used as chain extenders. When PEG structures are analyzed as monomers, PEG600 has the highest viscosity because it has the largest molecular weight, while PEG200 is the most fluid. On the contrary, as seen in Figure S1, when they were used in PU synthesis, PEG200 formed a more viscous pre-polymer as it has more dense cross-link points than the PEG600-based pre-polymer. The injectability of the final polymers was seen qualitatively in line with this theory. Free −OH ends were left at different rates (20, 30, and 40%) in the synthesized PUs. The reason for this is to synthesize free double bond-end PU-As by attaching acrylate monomer to these −OH ends. It constitutes the second and main cross-linking mechanism. The final PU-As were observed in accordance with the above theory, and depending on the cross-link frequency, the hardest structures were determined as the PEG200 containing polymers, while the most flexible ones were found to be PEG600. Additionally, PEG600-based polymers reacted relatively late and cured more slowly. For this reason, curing was determined as 5 min, and no monomer residue was ensured. The last cross-link mechanism consisted of the mussel-mimetic part. This mechanism was preferred to aid adhesion under water (the moist environment of tissues) rather than providing an extra cross-link point.

### 3.1 Structural and Qualitative Changes in Synthesis and Curing Steps

**Figure 2:**
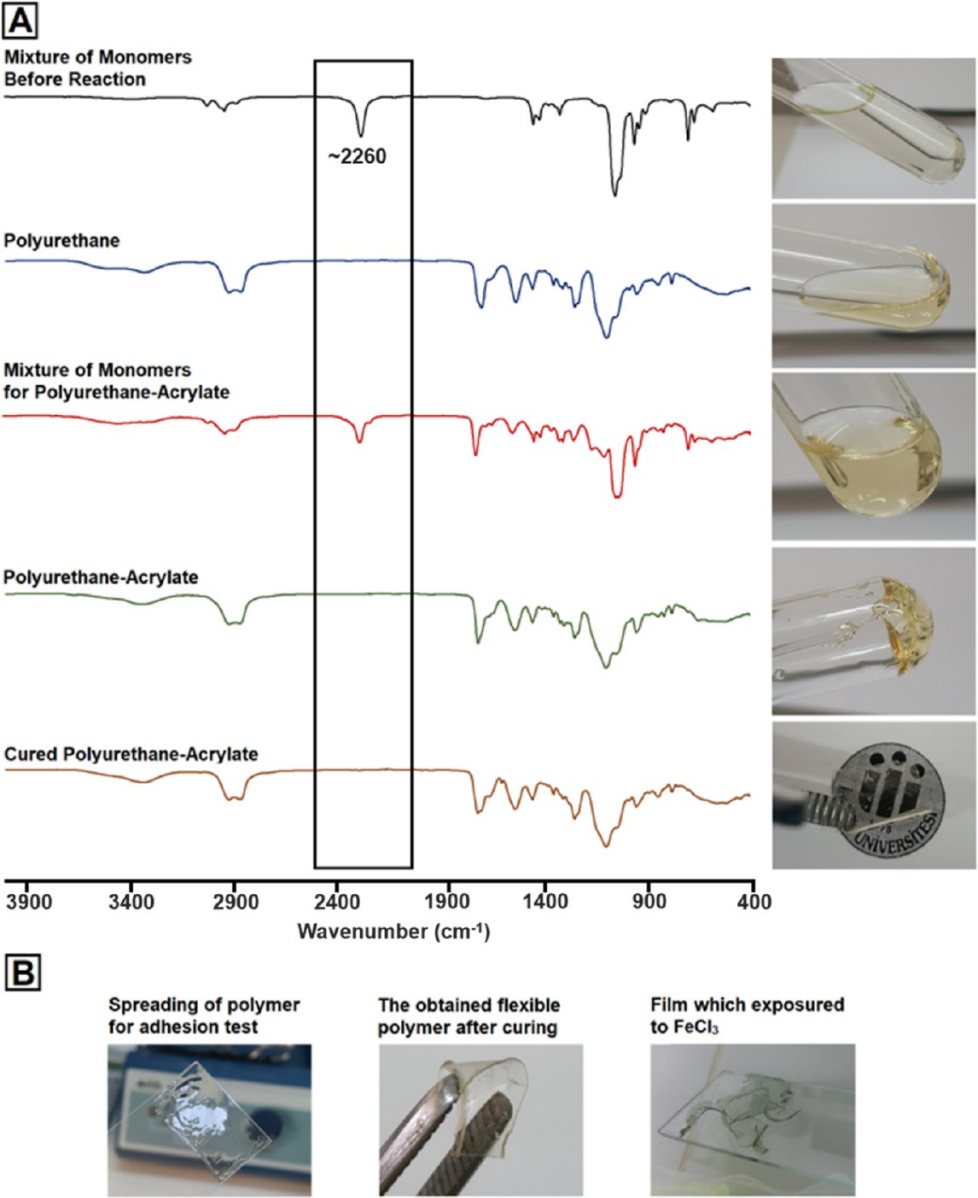
Structural and qualitative changes of polymers in each step during general synthesis and curing phase.

FTIR spectrum taken by mixing isocyanate and polyol source monomers before PU synthesis is shown in Figure 2A. As seen in the black spectrum, there is a large free isocyanate peak at ∼2260 cm^–1^.^39^ Qualitatively, a solution with a very light viscous liquid appearance was obtained. After being reacted for 24 h in an inert argon environment, the viscosity increased due to formation of PU structure, and the free isocyanate peak disappeared in the blue FTIR spectrum. Additionally, the color of the solution darkened slightly. Then, when 2-isocyanoethoethyl methacrylate was added to the structure, the free isocyanate peak was seen again in the same wavelength (red spectrum). After approximately 6 h of reaction in the inert atmosphere (green spectrum), the isocyanate peak disappeared again and a double bond functional polymer was obtained. When its solvent was evaporated, as shown in Figure 2A, a gel-like polymer was obtained. In the last stage, after curing with dopamine methacrylamide and Irgacure-2959 under UV light for 5 min, a flexible but completely solid form product was obtained (brown spectrum). Besides, when treated with FeCl_3_, the green color was formed due to the Fe–dopamine complex (Figure 2B).^51^

### 3.2 Characterization of Final Adhesives

The protein used in the study is sericin, a natural silk protein. Sericin is a high-molecular-weight, water-soluble protein derived from silk cocoons. It acts as an adhesive joining two fibroin filaments in order to form silk yarn. Sericin has high water binding capacity with high hydroxy amino acid content (about 46%).^52^ In the study, using the high hydroxyl content of the sericin, it was easily included in the PU structure and provided durability. PU structures containing sericin were also diversified using PEG200, PEG400, and PEG600. In this way, bone glue, which provides optimum mechanical and biocompatibility properties, has been obtained. The characterizations (FTIR, TGA, DTA, DSC, SEM, and AFM) of the PU-As are provided in the “Supporting Information” section.

### 3.3 Measurement of Free Hydroxyl Number of PUs

In the study, ASTM E1899-08 potentiometric titration standard method was used to determine the free hydroxyl (−OH) groups in the synthesized PU structures. Determination of free −OH numbers in PUs was important in terms of synthesizing double bond functional pre-polymers by interacting with 2-isocyanatoethyl methacrylate agent. The obtained data were plotted as % potential difference versus titrant volume (mL), and the free OH value in the formulations was given as mg potassium hydroxide (KOH)/g polymer. The results are provided in Figure S28. Free-OH numbers for HMDI-SER-P400-20, HMDI-SER-P400-30, and HMDI-SER-P400-40 samples were found as 127.257, 134.529, and 141.801 mg_KOH_/g_sample_, respectively. These data have been used to transform PUs into pre-polymer (double bond functional PU-A) formulations. Increasing amount of OH in theory with PEG ratio in PU formulations was confirmed by these results. However, the difference in hydroxyl number among the PUs was lower than expected even at high conversions of the monomers in the experiment. These results strongly suggest that most of the hydroxyl groups formed are trapped in stable hydrogen bondings and therefore unavailable for the reaction with isocyanates.^53^

### 3.4 *In Vitro* Adhesion Strength Analysis

In adhesion strength analysis test, our aim was to find the best tissue adhesive, rather than measuring the effects of adhesives on the target tissue. Therefore, we used glass slides in a dry environment to find the best adhesive, as the adhesive strength will decrease and the standard deviation will increase in the aqueous system. Instead, we performed *ex vivo* compressive strength test on the best adhesive in a humid environment (see Section 3.5). Furthermore, in the literature, aluminum substrates are generally used for *in vitro* measurement of adhesive strength, while glass substrates are used in UV-curable adhesives.

In the preliminary studies, the designed adhesive was ∼70% cured in about 1–3 min and reached the maximum strength in about 3–5 min. However, according to the amount of glue applied in the *in vivo* experiments, we did a 5 min curing because the monomer remaining in the tissue may cause toxic effects. Furthermore, as seen in histological and biochemical results of *in vivo* tests, there was no visible inflammation in the adhesive groups (see Section 3.9 and 3.10). Hence, it can be said that the UV light applied for 5 min did not cause toxicity in the tissues. In addition, because open heart surgeries take long hours, 5 min is not considered a long time in these operations.

**Figure 3:**
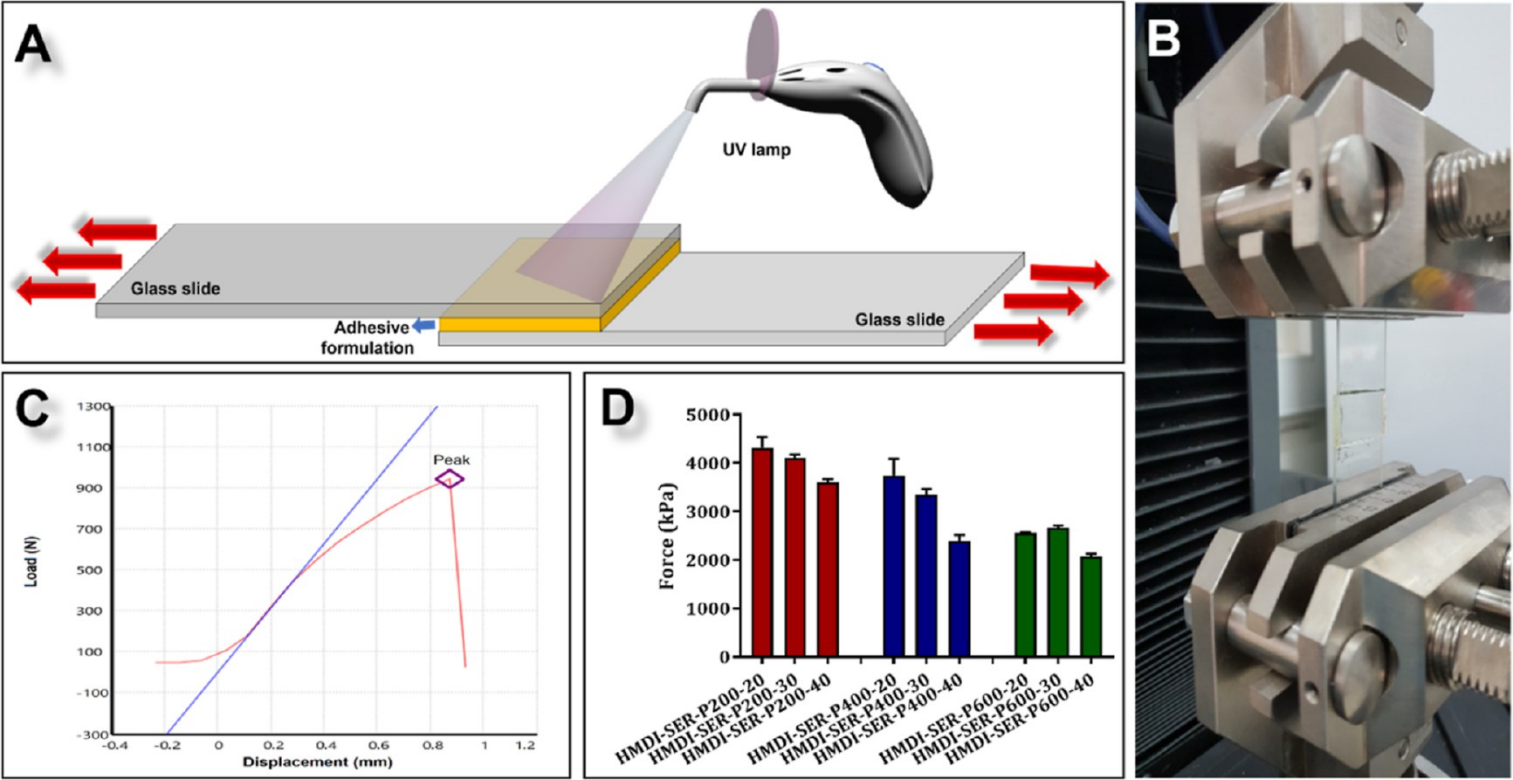
(A) *In vitro* lap shear adhesion test mechanism. (B) PU-As with glass slides placed in tensile device for adhesion test. (C) Measurement diagram taken as a result of the test. (D) Adhesion test results of the formulations.

The adhesion process showed in Figure 3A,B is described in the Experimental Section. The results are given in kPa (Table S1, Figure 3C,D). According to our results, the highest adhesion values were observed in the 20, 30, and 40% hydroxyl content of HMDI-SER-P200 as 4322.1 ± 214.9, 4108.7 ± 72.2, and 3607.5 ± 60.4 kPa, respectively. As the PEG molecular weight increased, it was observed that the adhesion strength decreased statistically significantly (*p* < 0.05). The reason for that as we move from the samples containing PEG200 to those containing PEG600 is that the physical flexibility of the material increased and a soft structure formed during the 5 min curing period. However, the adhesion strength decreased from the samples containing 20% hydroxyl to those containing 40%, but it was determined that these decreases were not significant (*p* > 0.05). The increase and decrease values in the adhesion strength were highly dependent on the PEG molecular weight. In addition to the adhesion differences due to cross-linking, the basic adhesion mechanism of the formulations is due to the polar nature of the PU structure. Due to the free −OH, −NH_2_, −SH, and −COOH and urethane groups in the structure, high adhesion strength can be obtained by making strong secondary interactions with different surfaces.^54^ Short curing time is also important in surgical operations. PUs are adhesives with high adhesion strength but require a long curing time.^54^ By binding the acrylates to the PU structure, curing can be achieved in a very short time (∼3–5 min) with UV light.^55,56^ In this study, the high adhesion strength of PUs and the fast curing properties of acrylates were combined. Furthermore, chelating was determined as the second curing mechanism in the polymer, with the addition of catechol units creating a mussel-mimetic zone. High adhesion strengths have been achieved with all these mechanisms.

At this stage of the study, HMDI-SER-P200-20-AC samples with the highest adhesion strength were selected from 9 synthesized PU-A formulations, and advanced biochemical characterization experiments were carried out.

### 3.5 *Ex Vivo* Compressive Strength Analysis

**Figure 4:**
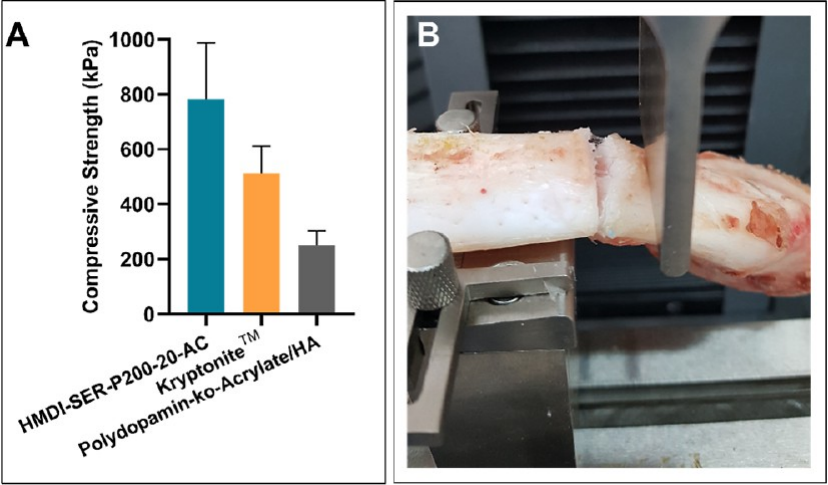
*Ex vivo* compressive strength results of HMDI-SER-20-AC, Kryptonite,^3^ and polydopamine-*co*-acrylate/HA.^3^

**Figure 5:**
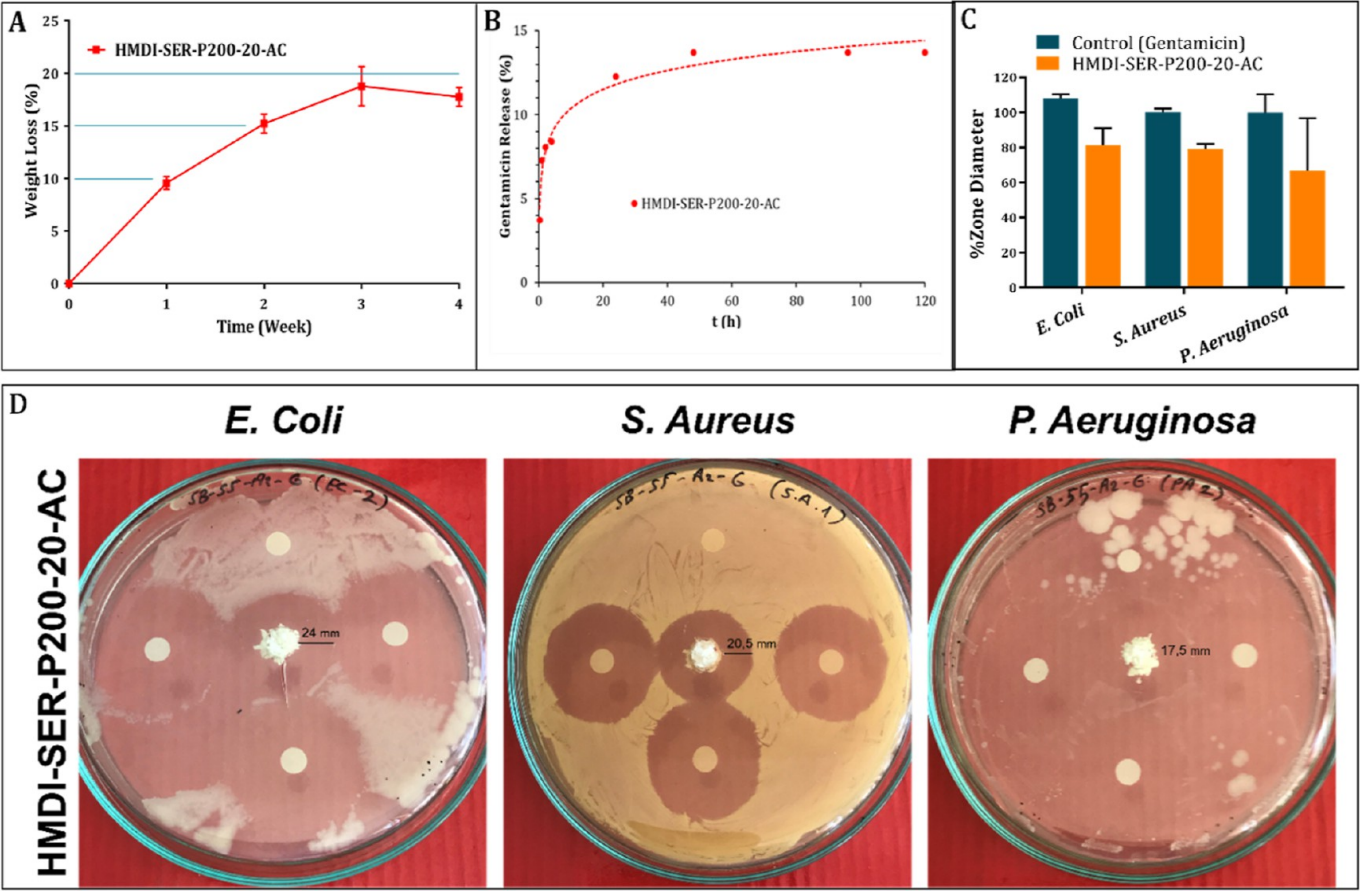
For the adhesive formulation (HMDI-SER-P200-20-AC); (A) biodegradability, (B) Gentamicin release, (C) % zone diameters on *E. coli*, *S. aureus,* and *P. aeruginosa* bacteria compared to Gentamicin, and (D) antibacterial properties and zone diameters on *E. coli*, *S. aureus,* and *P. aeruginosa* bacteria.

*Ex vivo* compression strength test was performed with bovine rib bone and compared with the literature data of Kryptonite^3^ and polydopamine-*co*-acrylate/hydroxy apatite (HA)^3^ used in sternal closure (Figure 4). According to the results, the HMDI-SER-P200-20-AC formulation showed a compressive strength of 783 kPa, while Kryptonite and polydopamine-*co*-acrylate/HA showed the compression strength as 514 and 215 kPa, respectively. These values proved that HMDI-SER-P200-20-AC formulation was comparable to the literature and showed higher compressive strength. Furthermore, the failure mechanism of the adhesive occurred as cohesion failure for the *ex vivo* test as seen Figure 5B and adhesion/cohesion failure for the *in vitro* adhesion strength test. As a result of this failure, it is concluded that the sternum can be opened easily where reopening is required during sternal closure.

### 3.6 Biodegradability, Gentamicin Release, and Antibacterial Activity Studies

The biodegradation property plays an important role in the design of tissue adhesives. The adhesives do not require to be removed or replaced in cases of infection especially after sternal surgery. However, degradation must also be slow because sternal recovery requires a long time. In our study, degradation was obtained relatively slow as 18% after 4 weeks in PU-A structure because it was cross-linked at a high level (Figure 5A).The results were at the highest level in the 1st week. After being completely resorbed, which takes relatively long time, the resultant fibrous tissue was strong enough to protect the intrathoracic organs.^57^ Methacrylate derivatives are used as bone glue in the literature, but their use is limited due to the lack of biodegradability.^58^ In other bone adhesives, bioactive glass was about 20%,^59^ isocyanate-terminated hyperbranched block copolymer was about %10,^60^ and polylactide methacrylate/tricalcium phosphate was 20–40%^61^ biodegradable. Therefore, our formulation is within the biodegradability limits of bone adhesives.

Gentamicin is the most commonly used antibiotic to prevent possible infections after sternal surgery. In the literature, collagen–Gentamicin sponges have been used to prevent local infection of the sternal region after being covered with steel wires.^62−66^ For this reason, 2.5% Gentamicin was doped to the adhesive formulation for local release. The results are given as cumulative Gentamicin release in Figure 5B. According to the results, releasing ratio was around 12% after 24 h.

Post-sternal surgery infections are mostly caused by *S. aureus*. Gentamicin is primarily used to prevent Gram-negative infection but also shows activity against some Gram-positive bacteria such as *Staphylococcus* species. Absorption of Gentamicin into the adhesive may assist in localized delivery at a higher concentration and may reduce systemic toxicity. In the literature, Gentamicin-collagen sponges are used for local antibiotic effect, and it has been reported that these sponges have no adverse effects.^64^ Therefore, Gentamicin was absorbed into the adhesive formulation to reduce systemic toxicity. The agar disc diffusion method was used to determine the antimicrobial activity of the adhesive. *E. coli* (ATCC 25922; Gram−), *P. aeruginosa* (ATCC 27853; Gram−), and *S. aureus* (ATCC 23235; Gram+) were used. Disc diffusion method images containing antibacterial activity results of HMDI-SER-P200-20-AC formulation are given in Figure 5D and % zone diameter data are given in Figure 5C. It was determined that the adhesive had an antibacterial effect similar to that of the pure Gentamicin. Gentamicin-free sterile discs were placed in each Petri dish as control, and no zones were observed around the discs. As seen in Figure 5D, the formulations showed around 80% activity compared to Gentamicin. Especially the zone diameters in *S. aureus* bacteria were very evident. Kimna et al. absorbed 0.2% Gentamicin into the zein-based electrospun mats, and they obtained 42 and 43% antimicrobial activity for *E. coli* and *S. aureus*, respectively.^67^ Sohail et al. investigated the antimicrobial effect of a lyophilized extracellular matrix envelope, hydrated in 40 mg/mL Gentamicin, on methicillin-resistant *S. aureus*, *E. coli*, *P. aeruginosa*, and *Staphylococcus marcescens*. According to their results, the envelope had killed all of the bacteria after 12 h.^68^ Our results showed that Gentamicin concentration in the adhesive was enough to supply antimicrobial activity.

### 3.7 *In Vitro* Biocompatibility Studies

**Figure 6:**
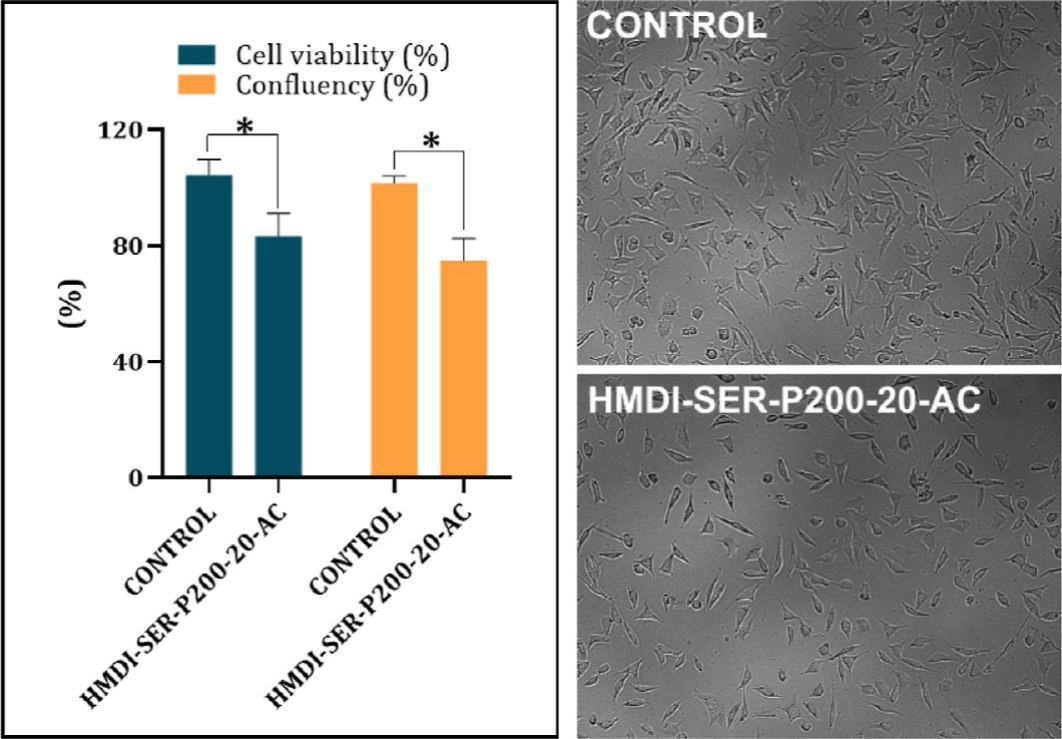
Biocompatibility results of HMDI-SER-P200-20-AC, * represents *p* < 0.05 (left); L-929 cell images (right).

In the biocompatibility test of HMDI-SER-P200-20-AC, formulation carried out by indirect method and mus musculus type mouse fibroblast cells (L-929) were used. As shown in Figure 6, the formulation showed approximately 83% (left) cell viability, and the difference between control and the adhesive formulation was significant (*p* < 0.05). Confluent ratios (*n* = 8) of the cells treated with the formulation were on the right. It was calculated by the inverter-based counting system from random wells. Although no significant difference was observed (*p* > 0.05), the results were parallel with the MTT. According to the cell morphology images on the far right in Figure 6, the formulation decreased the number of capillary extensions of the cells but did not show any change in overall morphology. The viability level here can be interpreted as a decrease in proliferation compared to control. In the MTT test, the material is considered non-cytotoxic if the percentage of the viable cell is greater than 70% of the untreated control according to ISO-10993-5. The results showed that the adhesive has sufficient biocompatibility.

### 3.8 Determination of Effectiveness of the Adhesive Formulation in Sternal Closure Surgery

Sternal closure using wire cerclage has remained the standard of care. Bone instability can occur after median sternotomy and cause significant morbidity, including sternal dehiscence, nonunion, and infection. The incidence of sternal deterioration is between 2 and 8%, and this destructive complication can cause death or permanent patient morbidity. Therefore, the number of steel wires that cause infection should be reduced. Also, where reopening is required, it is essential to close the sternum with easily removable materials. The most tried product as an alternative/supportive to steel wires is castor oil-based Kryptonite bone cement. Although many studies have stated that Kryptonite is safe, reliable, effective, and quick, it is no longer available on the surgical market.^13,14,16^ Another product developed as an alternative/supportive to steel wires is the hyperbranched poly (amino ester)-based nanocomposite adhesive reported by Zhang.^3^ Although the adhesive is reported to show good adhesion and mechanical properties, it does not meet the exact results as it has been tested in an *ex vivo* sternal model.

**Figure 7:**
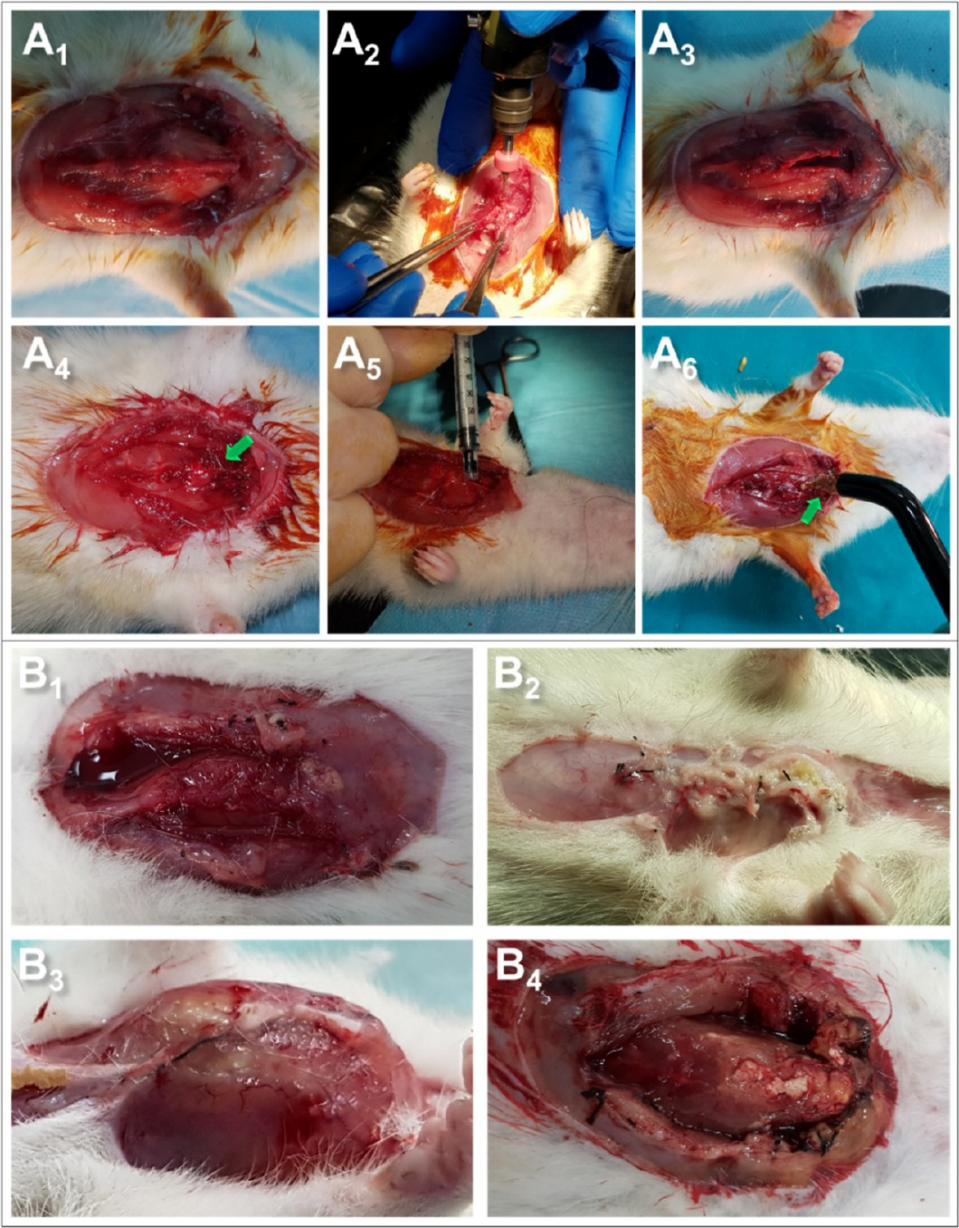
(A) Sternal closure surgery operation steps. (A_1_) general structure of the thorax, (A_2_) opening the sternum with the dental drill, (A_3_) opened sternum, (A_4_) closing the sternum with steel wire, (A_5_) closing the sternum with the commercial embolizing agent (cyanoacrylate), and (A_6_) closing the sternum with the synthesized adhesive formulation. (B) Thorax images of rats 1 week after sternal closure. (B_1_) Control (sternum not opened), (B_2_) cyanoacrylate, (B_3_) stainless steel wire, and (B_4_) HMDI-SER-P200-20-AC.

In our study, for the sternal closure operation, the thorax of 24 Wistar male rats (Figure 7A_1_) was opened and the sternum was divided into two longitudinally with the help of loop glasses and dental drill (Figure 7A_2_ and Figure 7A_3_) and closed with stainless steel wire (Figure 7A_4_) cyanoacrylate (Figure 7A_5_) and the adhesive (HMDI-SER-P200-20-AC) (Figure 7A_5_). The adhesive formulation group was exposed to UV light for 5 min after the adhesive was applied (Figure 7A_6_). After 7 days, the animals were sacrificed and histological and biochemical analyses were performed by removing the surrounding tissue. When the thorax was opened at the end of the specified period, there was no inflammation in the control group (Figure 7B_1_), while some animals had very high inflammation in the cyanoacrylate (Figure 7B_2_) and steel wire groups (Figure 7B_3_). On the other hand, there was no visible inflammation in the adhesive formulation (Figure 7B_4_). The pictures show that there is a dramatic difference in inflammation between our formulation and that of the other groups. In the adhesive groups, steel wires were placed in the head and end of the sternum, but no inflammation was observed in these groups, although they contained steel wires. One of the reasons for this was that the adhesive penetrates between the wire and the tissue, eliminating the inflammation risk of the wire. Another reason was to prevent inflammation by reducing the number of steel wires. On the other hand, because the middle part of the sternum is softer, it is more susceptible to cutting by steel wire. It was thought that sealing this area with adhesive will also eliminate the risk of the sternal cut. Furthermore, considering the situations requiring reopening, after the animals were sacrificed, the sternum was divided into two with the help of a scalpel. While there was no problem in the adhesive formulation, it was difficult to separate the cyanoacrylate group due to its uneven adhesion. In steel wires, the process was much more difficult as it requires manual separation. In summary, removing the adhesive from the cartilage/bone tissue would be much easier than steel wires and cyanoacrylate adhesive when the sternum needs to be reopened.

### 3.9 Histological Analysis

**Figure 8:**
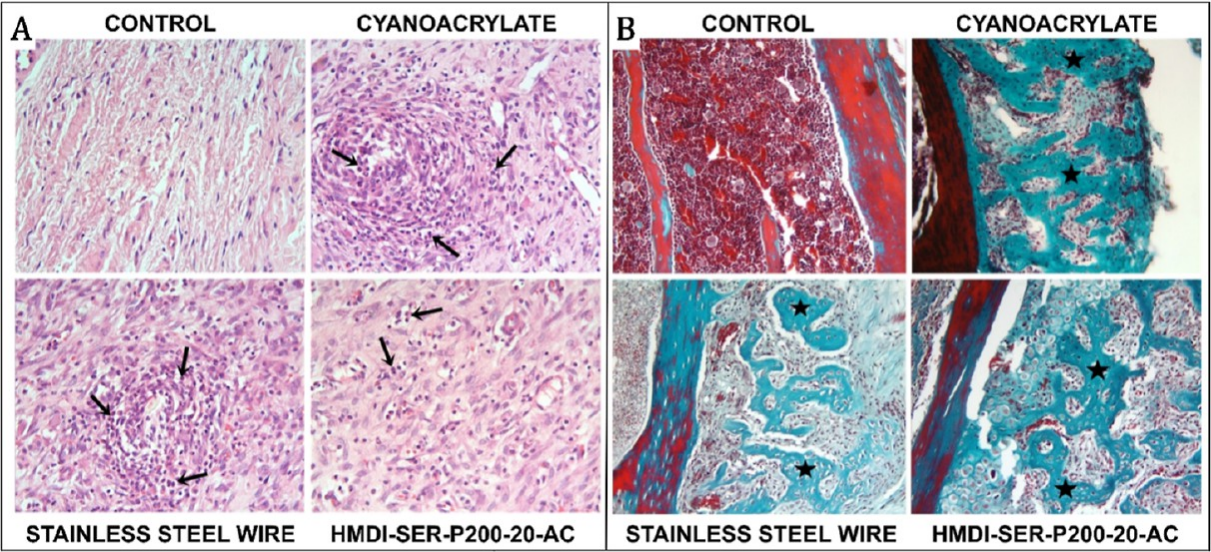
(A) Infiltrative cells (arrows) around the sternum attract attention in other experimental groups, except the control group. H–E; ×40; (B) developing bone trabeculae around the sternum in the other experimental groups except for the control group H–E; ×20.

In sections with the H–E staining method, endochondral ossification centers and developing bone trabeculae were observed around the original sternum, while large callus tissues were noted in more periphery tissues. A mild inflammatory reaction was observed in all experimental groups (Figure 8A). However, the infiltration severity observed in the steel wire and cyanoacrylate groups was found to be statistically significantly higher compared to the adhesive formulation (*p* < 0.05). It was noteworthy that in the sections applied in the MT staining method, full bone regeneration was not observed throughout the sternum incision, but endochondral ossification was similar in all groups and at similar levels (Figure 8B). Histological evaluation results are given in Figure S31A, and comparison results between groups (*p* values) are given in Figure S31B.

### 3.10 Biochemical Analysis

MPO and NO as the inflammation parameters in the tissues around the sternum and BUN and CR amounts in serum samples were determined, and it was checked whether there was a difference compared to the control.

**Figure 9:**
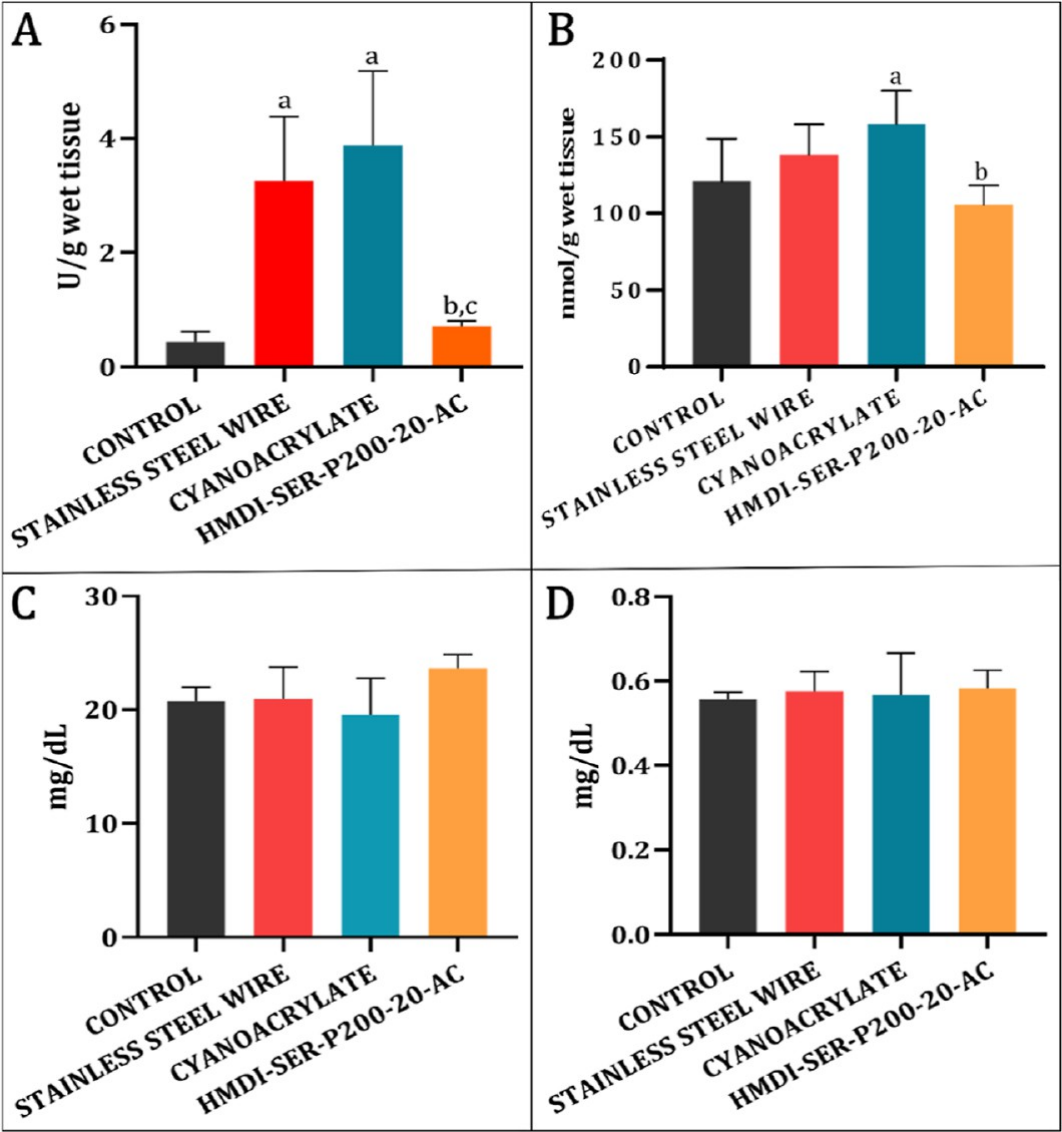
(A) MPO and (B) NO results in tissues taken from muscle circumference. Statistically significant (*p* < 0.05); (a) compared to the control, (b) compared to the cyanoacrylate, and (c) compared to the steel wire group. (C) BUN and (D) CR levels in blood samples taken after sternal closure.

MPO results in tissue samples are given in Figure 9A. According to the results, there was a statistically significant increase in the steel wire and cyanoacrylate groups compared to the control (*p* < 0.05). These results showed that both steel wire and cyanoacrylate adhesive caused biochemical inflammation around the sternum. However, in the developed formulation, close activity was obtained in the control group and inflammation of the formulation was significantly smaller than other groups (*p* < 0.05).

NO results in tissue samples are given in Figure 9B, and there was a statistically significant increase in the cyanoacrylate group compared to the control (*p* < 0.05). In the formulation, there was no statistically significant change in NO level compared to the control (*p* > 0.05). The reason for the low inflammatory response of the synthesized adhesives was the use of more biocompatible aliphatic isocyanates instead of aromatic and more toxic types and the use of fully biocompatible monomers such as PEG and sericin. In the literature, there were studies in which PU-based structures showed an inflammatory response close to control in acute toxicity process.^8^

In the study, BUN and CR levels were determined to evaluate kidney functions in serum samples during the biodegradability process of the adhesive formulation after sternal closure (Figure 9C,D). According to our results, BUN and CR levels were similar in all groups. These results showed that there was no irregularity in kidney function of rats, and it was in line with biocompatibility results.

## 4 Conclusions

In this study, we reported a biomimetic, biodegradable, biocompatible, antibacterial, and UV-curable PU-A tissue adhesive for sternal closure as supportive to wire cerclage. Using biocompatible monomers in the adhesive design promoted biocompatibility and biodegradability. The highest adhesion values were observed in the 20% hydroxyl content of HMDI-SER-P200 as 4322.1 ± 214.9 kPa. The adhesive was used in the rat model of sternal closure as a supportive to stainless steel wire in order to reduce operation-induced failure such as inflammation and interrupted sternum. Incorporation of Gentamicin by host–guest reactions with β-cyclodextrin provided local drug release and prevented the inflammation in the operation area in *in vivo* sternal closure model in rats. However, a significant increase in terms of inflammation was observed in cyanoacrylate and stainless steel wire groups. These results proved that the PU-A adhesive could be a good candidate for sternal closure as a supportive the wire cerclage.

## Supporting Information Available

FTIR, DTA, TGA, DSC, and NMR results; hydroxyl numbers of the formulations; SEM and AFM results; adhesion test results; and inflammation scores (PDF)
