# Supplementary material for: Fast Curing Multifunctional Tissue Adhesives of Sericin-Based Polyurethane-Acrylates for Sternal Closure
Source: ACS Appl Mater Interfaces. 2022 Sep 6;14(37):41819–33. doi: 10.1021/acsami.2c14078 (PMC9501797; doi:10.1021/acsami.2c14078)
Supplement: Supplementary file 1 — am2c14078_si_001.pdf [file am2c14078_si_001.pdf]

Supporting Information

Fast Curing Multifunctional Tissue Adhesives of Sericin-Based Polyurethane-Acrylates for Sternal Closure

Sevgi Balcioglu[1], Samir Abbas Ali Noma[2], Ahmet Ulu[3], Merve Göksin Karaaslan-Tunc[4], Onural Ozhan[5], Suleyman Koytepe[3], Hakan Parlakpinar[5], Nigar Vardi[6], Mehmet Cengiz Colak[7], Burhan Ates[3,*]

[1]*Sakarya University of Applied Sciences, Department of Medicinal Laboratory, 54000, Sakarya, Turkey*

[2]*Bursa Uludağ University, Faculty of Arts and Sciences, Department of Chemistry, 16059, Bursa, Turkey*

[3]*İnönü University, Faculty of Arts and Sciences, Department of Chemistry, 44210, Malatya, Turkey*

[4]*Selçuk University , Taşkent Vocational School, 42000, Konya, Turkey*

[5]*İnönü University, Medical Faculty, Department of Medicinal Pharmacology, 44210, Malatya, Turkey*

[6]*İnönü University, Medical Faculty, Department of Histology and Embryology, 44210, Malatya, Turkey*

[7]*İnönü University, Medical Faculty, Department of Cardiovascular Surgery, 44210, Malatya, Turkey*

***Corresponding Author:**

Burhan Ates, PhD

Biochemistry and Biomaterials Field, Inonu University, Department of Chemistry, 44210, Malatya, Turkey

E-mail; burhan.ates@inonu.edu.tr;

ORCID: https://orcid.org/0000-0001-6080-229X

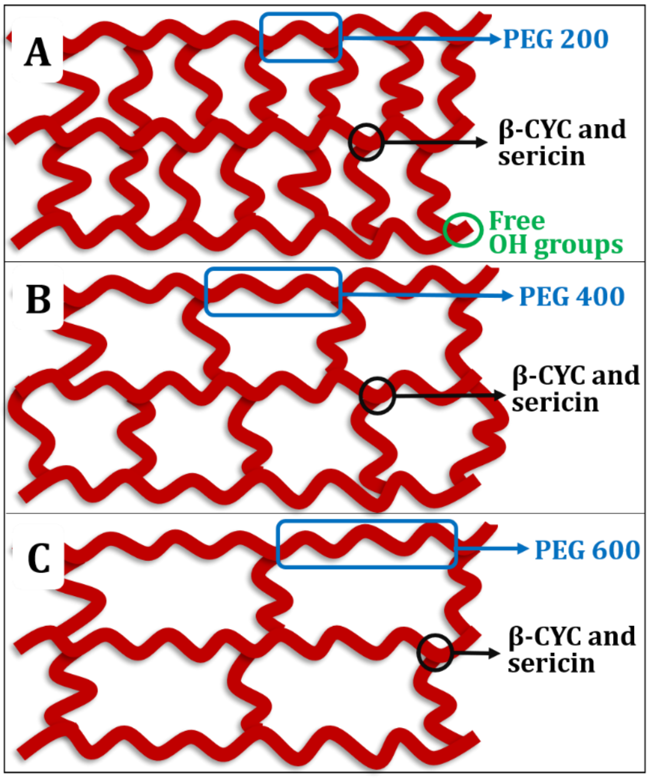

Figure S1. Crosslink frequency of PEG200, PEG400 and PEG600-based polyurethanes

Structural and Thermal Characterization

FTIR spectra of sericin-based adhesives containing 20% hydroxyl and PEG200 units (HMDI-SER-P200-20) were given in Fig. S2. In these spectra, all vibration peaks arising from the polyurethane structure were seen as well as the amide vibrations arising from the sericin. The C=O intense tensile vibration at 1711 cm^{-1}, the N-H tensile vibration at 3300 and 1458 cm^{-1} of the polyurethane structure and the aliphatic C-H tensile vibration at 2900-2800 cm^{-1} of the PEG structure were observed. Furthermore, C-O-C tensile vibrations caused by PEG and cyclodextrin structures were clearly seen at 1274 and 726 cm^{-1}. These peaks proved that the desired polyurethane structure was obtained clearly. According to the FTIR results, it can be said that acrylate groups were attached to the structure by UV-curing. After curing, increased methylene peaks and C-C tensile vibrations seen at 1616 cm^{-1} proved the polyurethane-acrylate structure [1-3]. The adhesive, which is in liquid form before curing, had the appearance of a solid, transparent

and colorless polymeric film after curing. This change proved that the crosslinking efficiency was high.

FTIR spectra of sericin-based adhesives containing PEG200 and 30% free hydroxyl (HMDI-SER-P200-30) were shown in Fig. S3. In the spectrum, –OH peak at 3439 cm^{-1}, –CH at 2884 cm^{-1} and –COO at 1720 cm^{-1} were observed. Also, NHCOO strain at 1694 cm^{-1}, -NH tension at 3319 cm^{-1}, and N-H bending at 1537 cm^{-1} were seen. These structures substantially proved that the desired polyurethane structure was formed. In the red spectrum to which the acrylate units were bound on the related polyurethane structure, it can be seen that the peak at 2265 cm^{-1} causing from free isocyanate groups of 2-isocyanatoethyl methacrylate disappeared. It proved that the acrylate group was included in the polymer structure. Also, C-H off-plane bending at 675 cm^{-1} originating from C=C double bond can be interpreted for the presence of acrylate structures. In the last spectrum, after the curing, this peak received a wider and band character. The expansion of the aliphatic C-H tensile vibration (2830-2950 cm^{-1}) proved that curing was achieved.[1–3]

In Fig. S4, the spectra of PEG200, sericin and 40% free hydroxyl containing polymers (HMDI-SER-P200-40) were shown. In this spectrum, basic polyurethane peaks were seen, similar to others. These are C=O (1720 cm^{-1}), N-H (1582 cm^{-1}) tensile vibration, C-N tensile vibration (1440 cm^{-1}), C-O-C tensile vibration (1240 cm^{-1}) peaks originating from the classical urethane structure. In addition, the absence of free isocyanate peaks in all spectra and the formation of these peaks proved that the urethane bond was established in the polymer. Another component was cyclodextrin groups in the structure. Due to these cyclodextrin groups, we can clearly see C-H tensile vibrations between 1000-1092 cm^{-1} in

all polymeric structures. The desired polymeric film formulation and increased aliphatic C-H stresses in cured systems showed that curing was clear.[1–3]

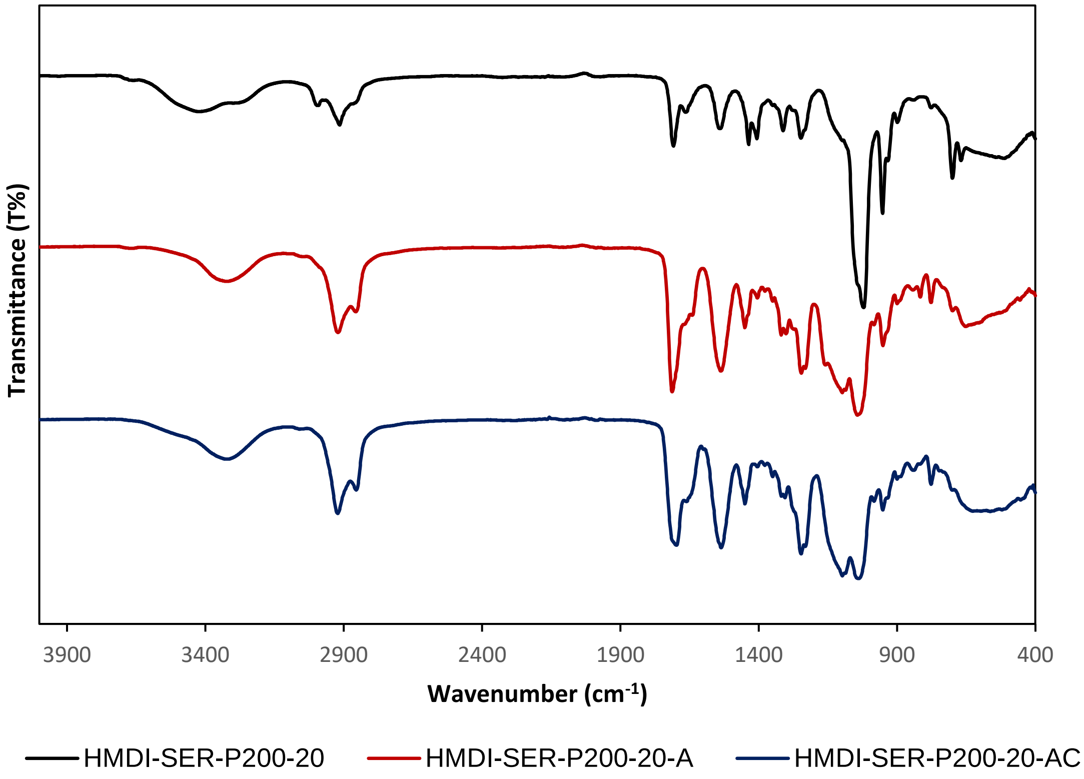

Figure S2. FTIR spectra of HMDI-SER-P200-20 based polymers

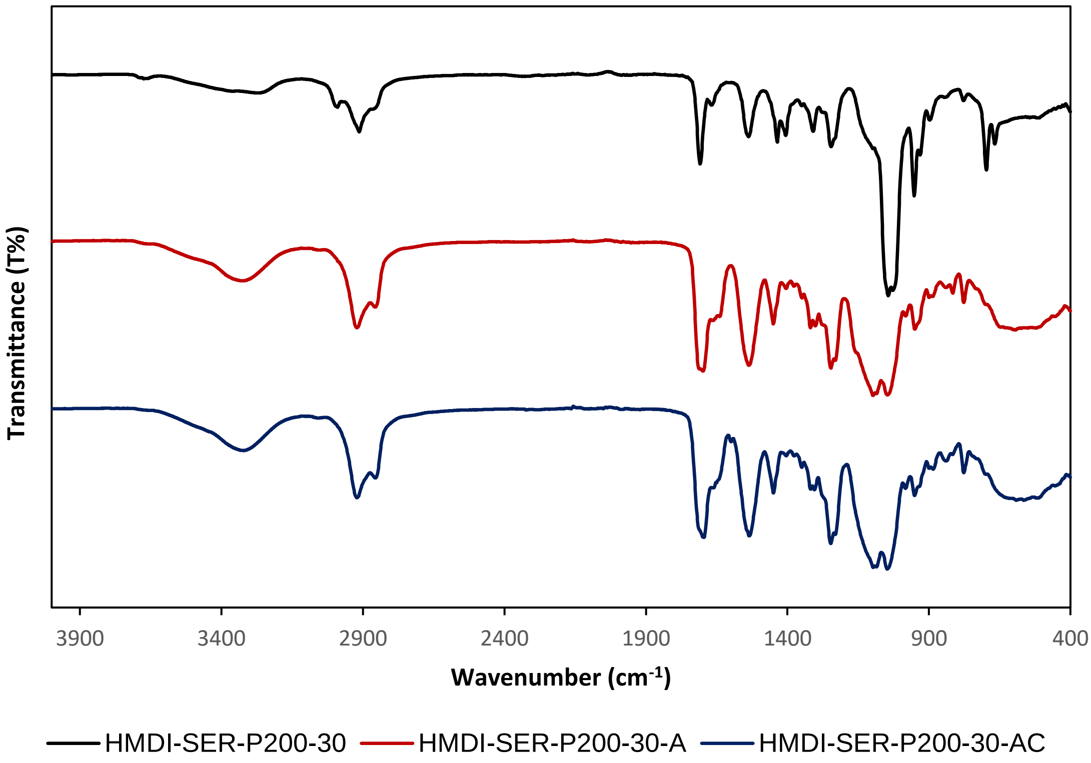

Figure S3. FTIR spectra of HMDI-SER-P200-30 based polymers

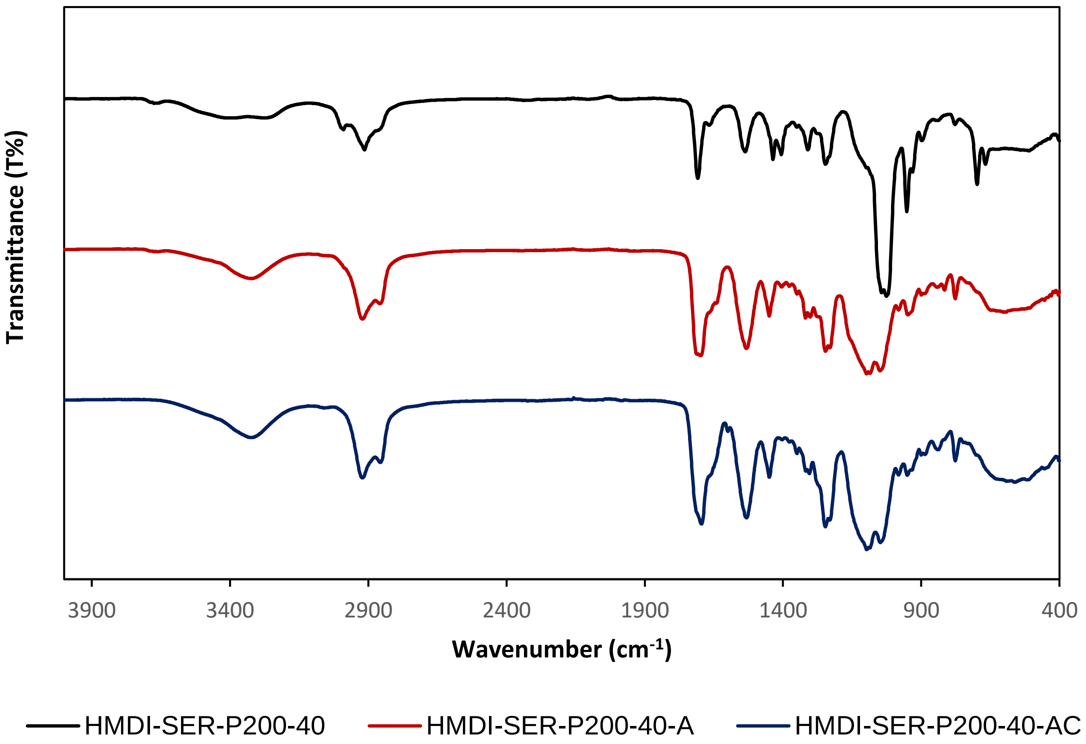

Figure S4. FTIR spectra of HMDI-SER-P200-40 based polymers

Tg values of polymers were seen as 16.21, 15.61 and 16.47 °C for HMDI-SER-P200-20, HMDI-SER-P200-30 and HMDI-SER-P200-40, respectively (Fig. S5). These values increased after curing to 20.91, 17.72 and 29.08 °C, respectively. This is due to the increased rigidity with increasing crosslink ratio after curing.[4] This interpretation was seen in accordance with the DTA thermograms shown in Fig. S6. DTA thermograms showed that the structural stability of the polymer increased as the structural cross-linking increased. Decomposition start temperatures were moved to a higher value after curing.

Fig. S7, Fig. S8 and Fig. S9 showed TGA thermograms of the polymers containing PEG200-sericin. There were three main mass losses in these thermograms. The first mass loss was between 80 °C and 110 °C, the second mass loss was between 180-320 °C and the final mass loss was between 320-490 °C, which proved that thermal stability increases with increasing cyclodextrin ratio because of the high crosslinking [4]. The first mass loss showed the removal of partial moisture in the polymer structure, the second mass loss showed the soft segment degredation and the last mass loss showed the thermooxidative degradation of the polymeric structure. In general, the first drop around 100 °C is due to structural humidity. For this reason, we see that the thermal stability of polymeric structures is about 180 °C. This value reaches higher temperatures through curing. These results showed that polymer structures are suitable for using within the body temperature.

In DSC, TGA and DTA results, PU presented generally a relatively poor thermal stability while enhanced thermal stability was observed by the chemical incorporation of acrylate in polyurethane acrylate (PU-A).[4]

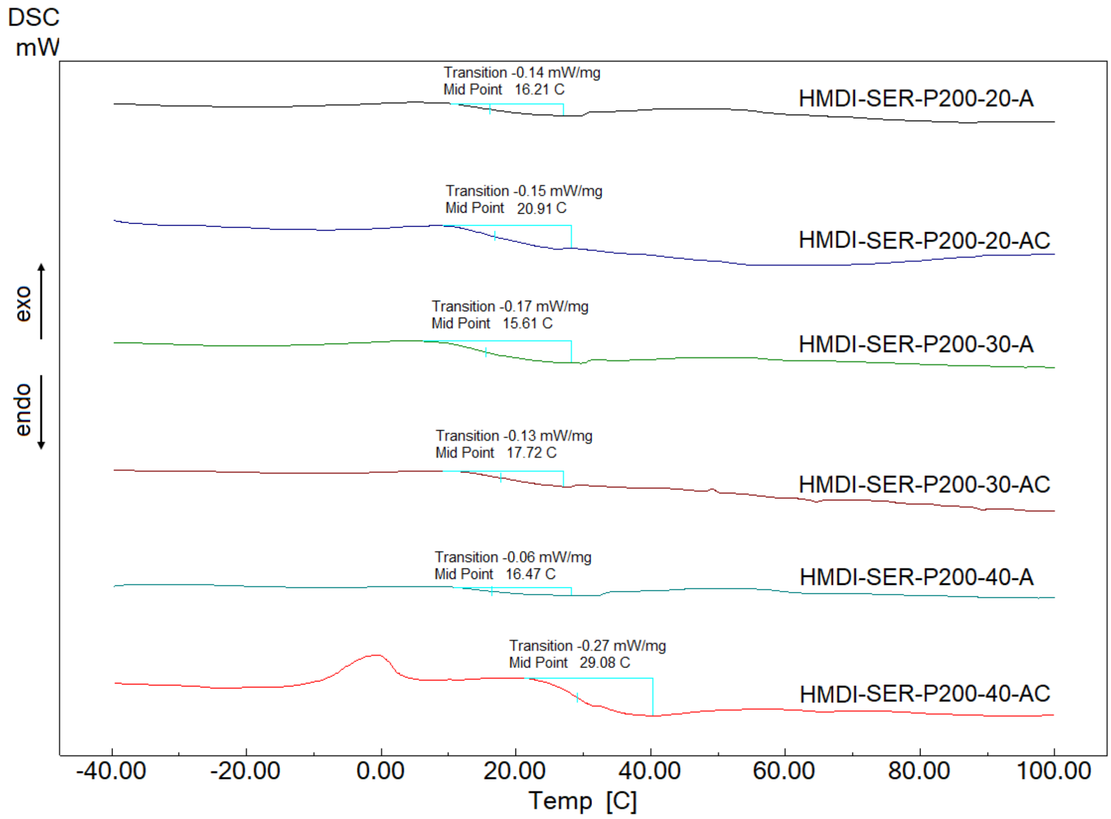

Figure S5. DSC thermograms of HMDI-SER-P200 based polymers

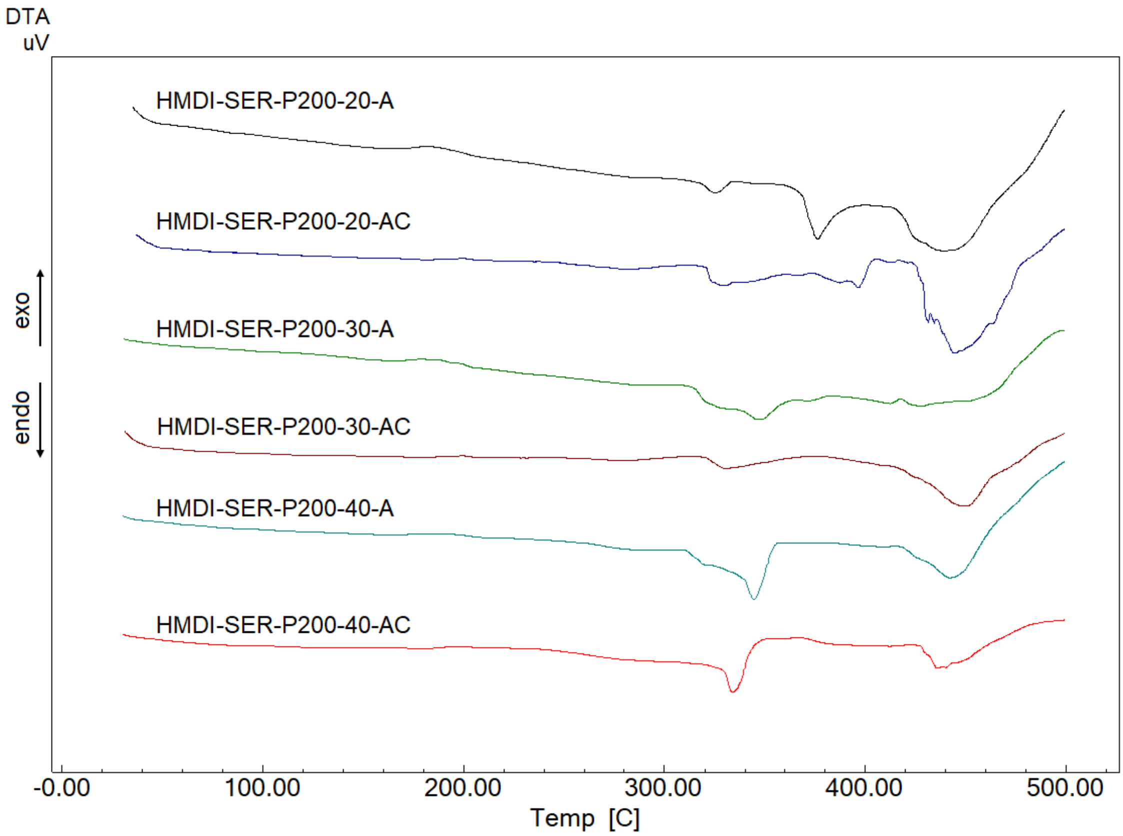

Figure S6. DTA thermograms of HMDI-SER-P200 based polymers

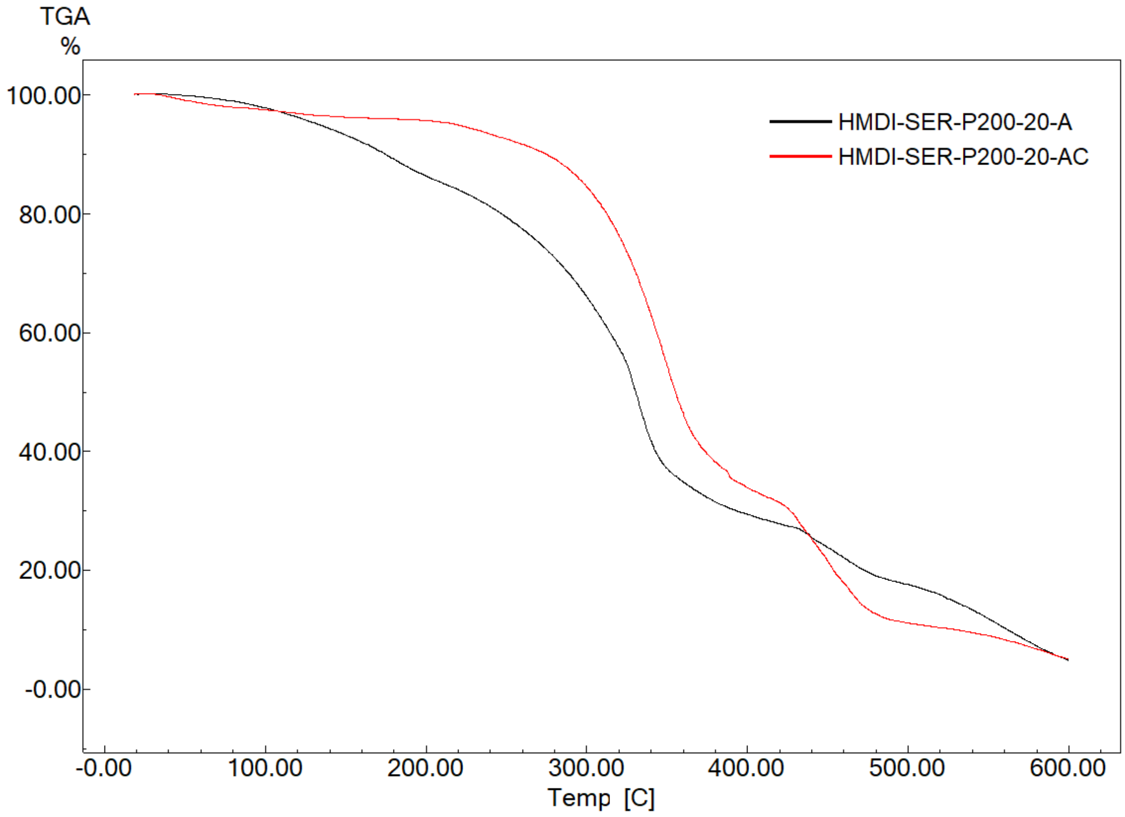

Figure S7. TGA thermograms of HMDI-SER-P200-20 based polymers

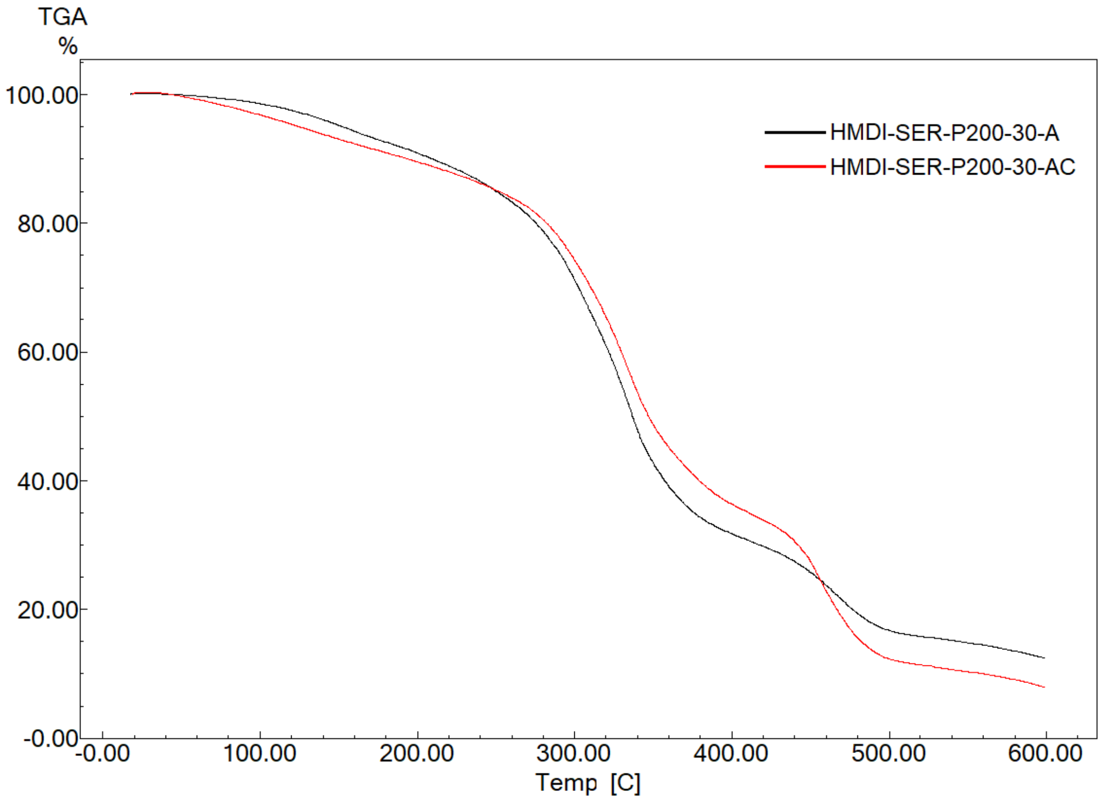

Figure S8. TGA thermograms of HMDI-SER-P200-30 based polymers

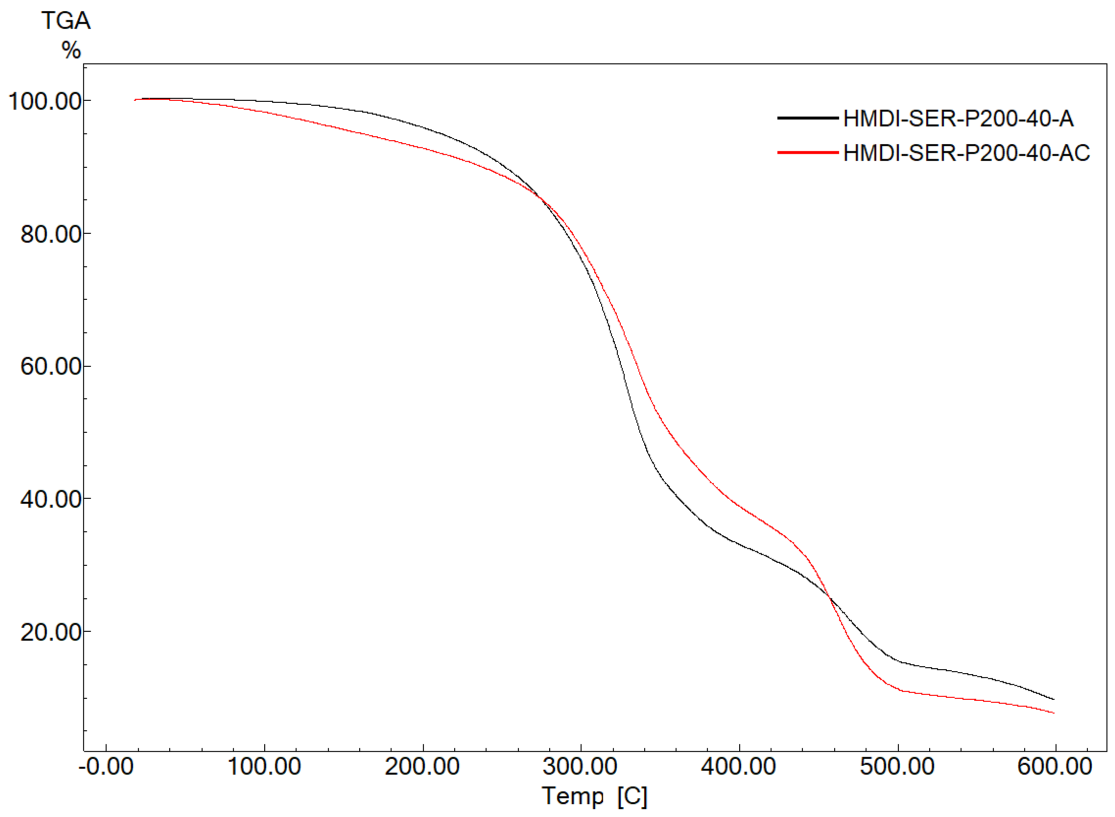

Figure S9. TGA thermograms of HMDI-SER-P200-40 based polymers

In the study, sericin-based adhesives carrying PEG400 units were characterized by FTIR. By increasing the PEG chain length, the applicability (spreadability), viscosity, structural flexibility, mechanical strength and Tg values of the polymeric adhesive were obtained in the desired ratio. When the PEG structure was changed from PEG200 to PEG600 in the polyurethane, polyurethane-acrylate and cured structures, a higher flexibility was obtained. At the same time, as a disadvantage, the applicability of the polymer and its solubility has decreased. The spectra were given in Figure S10, Figure S11 and Figure S12.

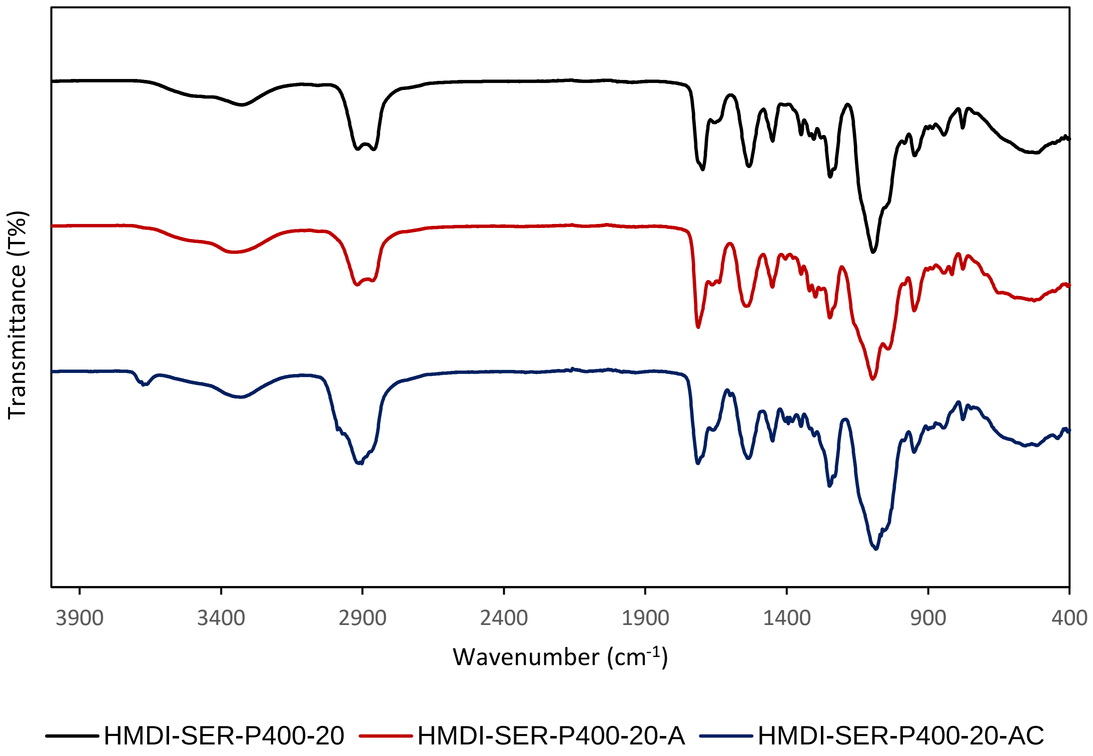

Figure S10. FTIR spectra of HMDI-SER-P400-20 based polymers

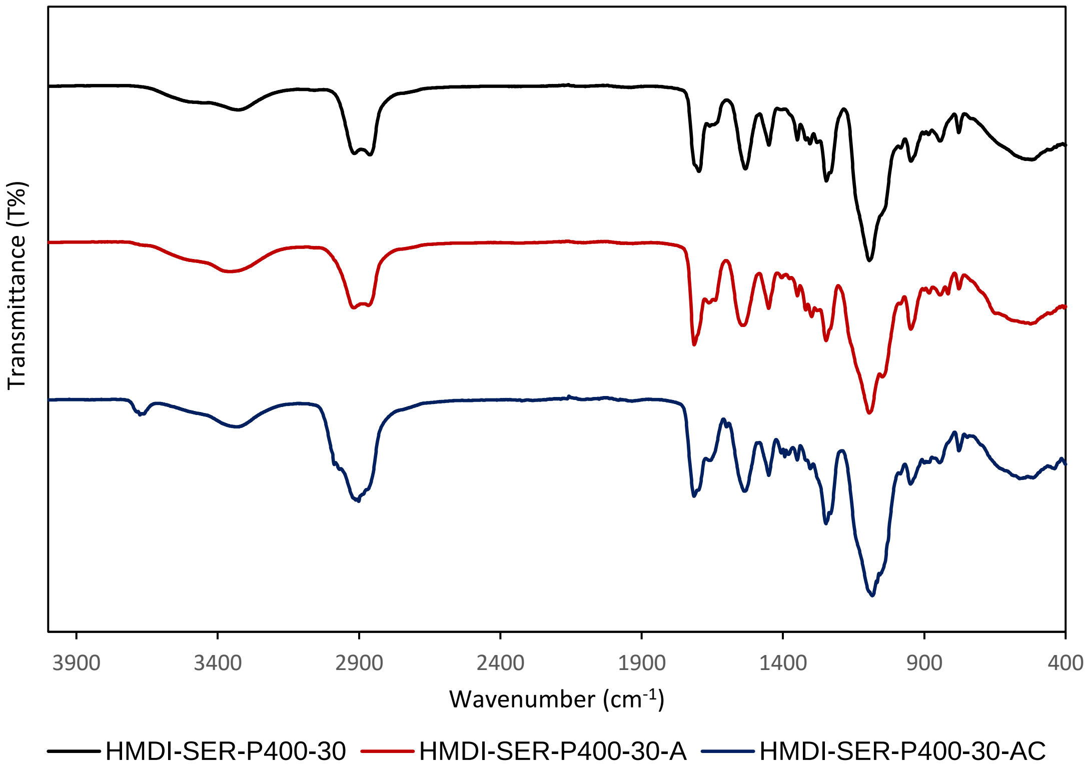

Figure S11. FTIR spectra of HMDI-SER-P400-30 based polymers

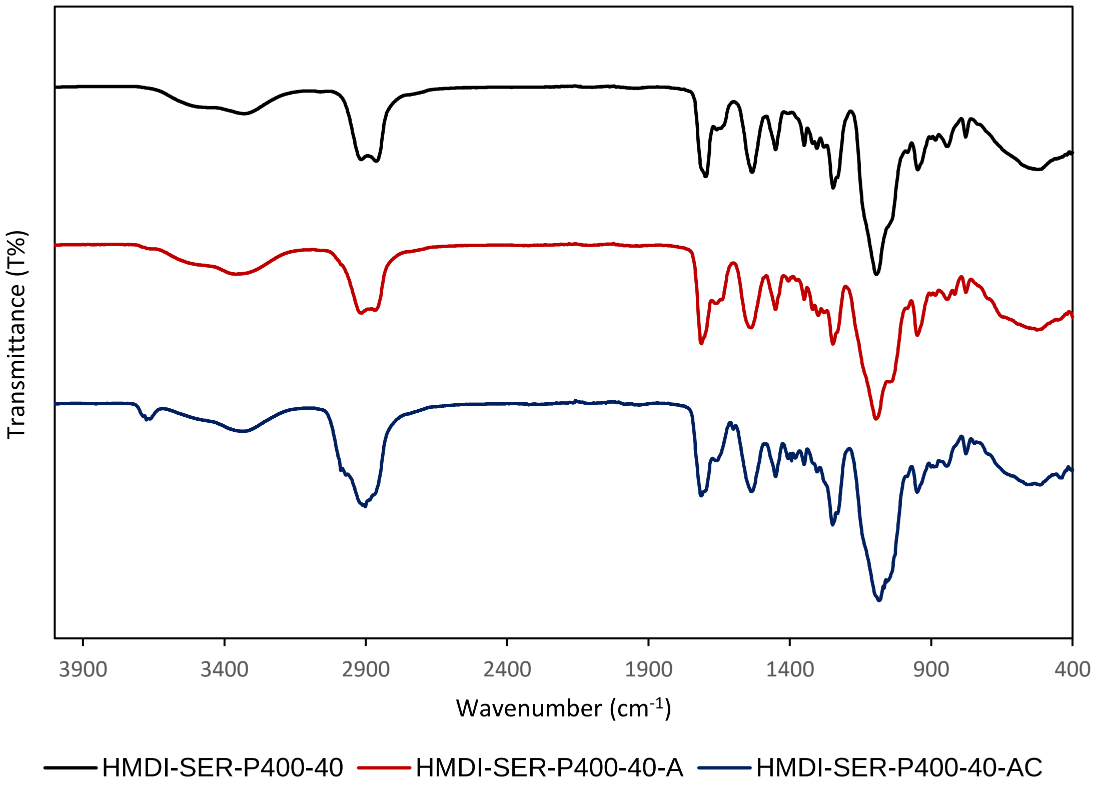

Figure S12. FTIR spectra of HMDI-SER-P400-40 based polymers

In the spectra given in Figure S10, Figure S11 and Figure S12, the formation of urethane bond and the loss of free isocyanate peak were observed. The absence of any peak around 2265 cm^{-1} in all polymers in Figure S10 is an indication that there is no free isocyanate group and the polyurethane structure is formed. Etheric bonds showing the formation of polymeric bond with sericin units were seen at 1274 cm^{-1}. The carbonyl tensile vibration at 1716 cm^{-1}, urethane bond N-H tensile vibration at 3200-3600 cm^{-1} and urethane bond C-N-O tensile vibration at 1584 cm^{-1} were seen. In the polyurethanes, the intensity of the etheric and methyl peaks increased due to the curing process.

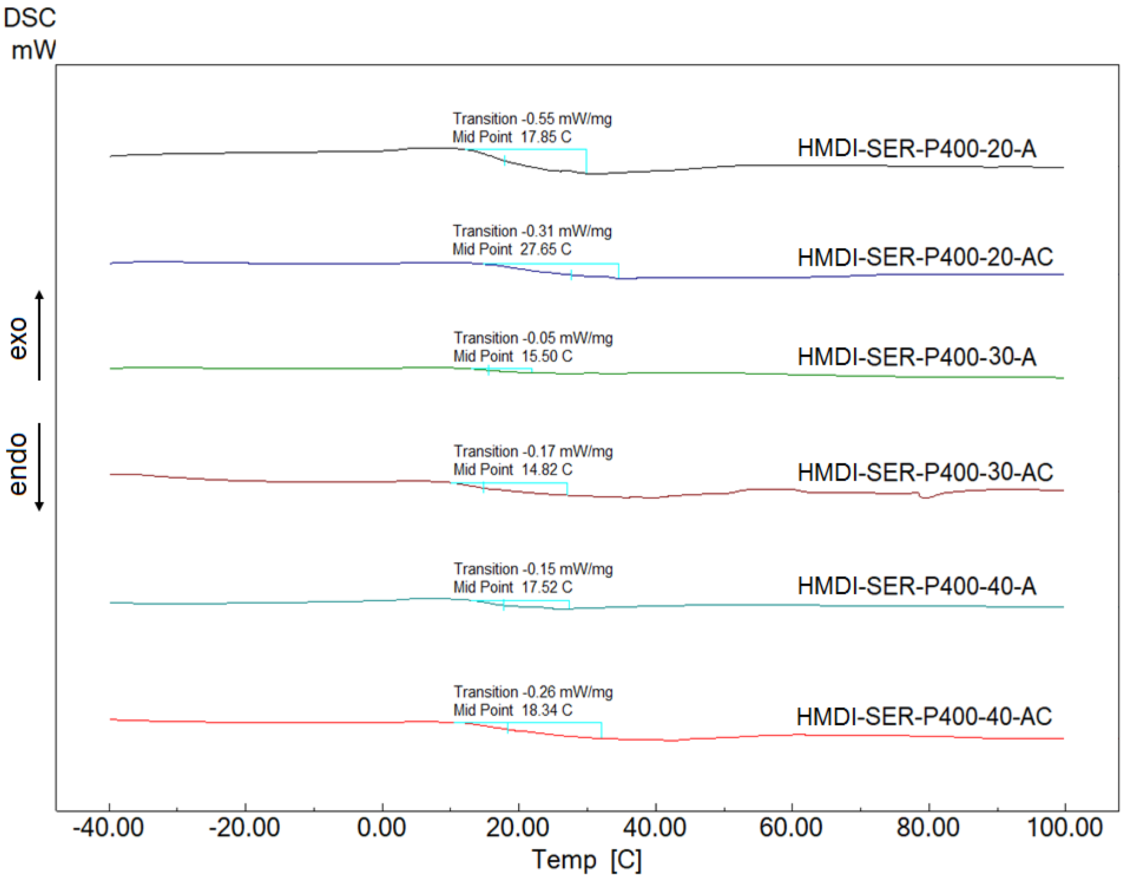

Figure S13. DSC thermograms of HMDI-SER-P400 based polymers

The changes in the general thermal properties of the PEG400-sericin polymers were examined by DTA, TGA and DSC analyzes. In Figure S13, DSC thermograms of the polymers containing PEG400-sericin were given. Tg values of PEG400-sericin structures were observed around 17 °C in these thermograms. This value indicated that the polymer was injectable and applicable in the body. In addition, this value increased with curing. OH groups originating from cyclodextrin were reduced and were crosslinked with PEG and diisocyanates. In addition, urethane groups also increased the crosslinking points. As a result of these cross-links, it becomes difficult for the polyurethane chains to approach each other, so a decrease in polymer resistances occurs. Due to this effect, Tg temperatures of polymers increased. DTA thermograms given in Figure S14 gave two exotherms to verify this finding. In these thermograms, an exotherm was seen due to the degradation of the polymeric binding units. Structural and thermal stability increased significantly with curing. TGA thermograms also supported these results. In TGA thermograms, first of all, 2% moisture removal was seen around 100-110 ° C. Later, a mass loss

was observed around 250-350 °C due to the degradation of the protein and PEG groups in the polyurethane structure. Finally, degradation of cyclodextrin groups and mass losses of thermoxidative degradation were observed in Figure S15, Figure S16, Figure S17 and Figure S18. In addition, as the structure was cured, the soft segment degradation and degradation initial temperature increased.

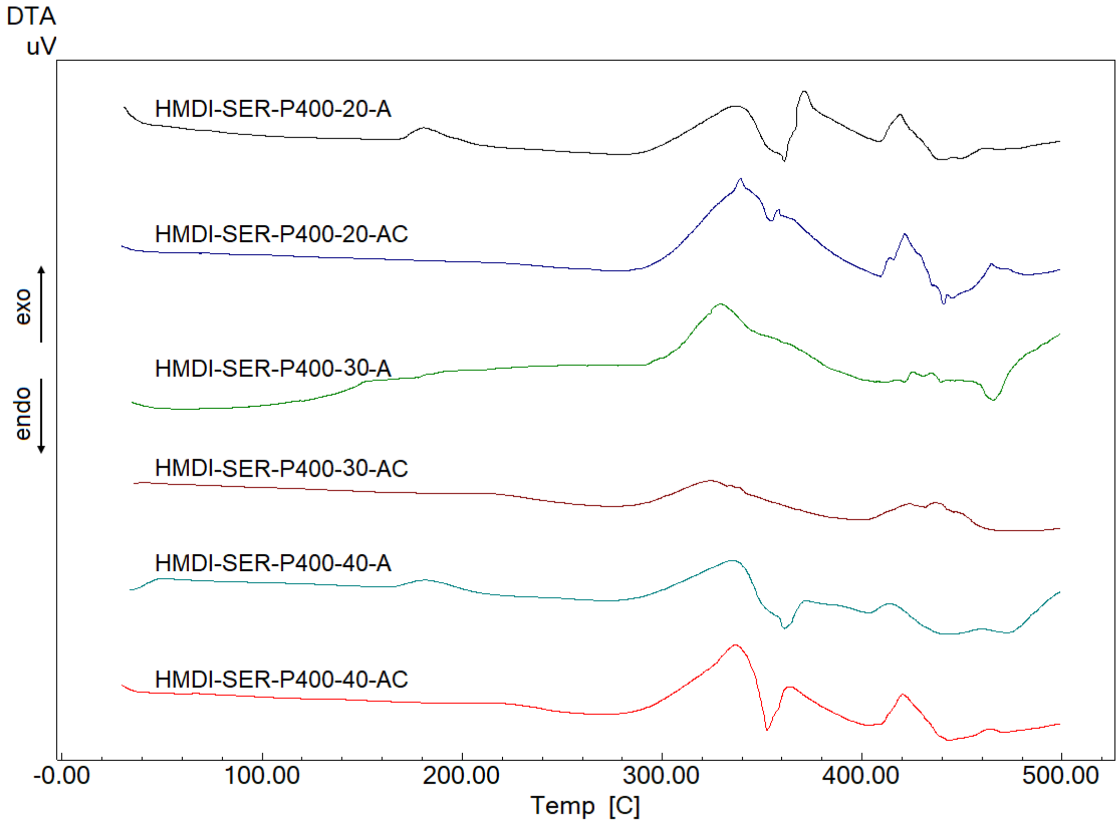

Figure S14. DTA thermograms of HMDI-SER-P400 based polymers

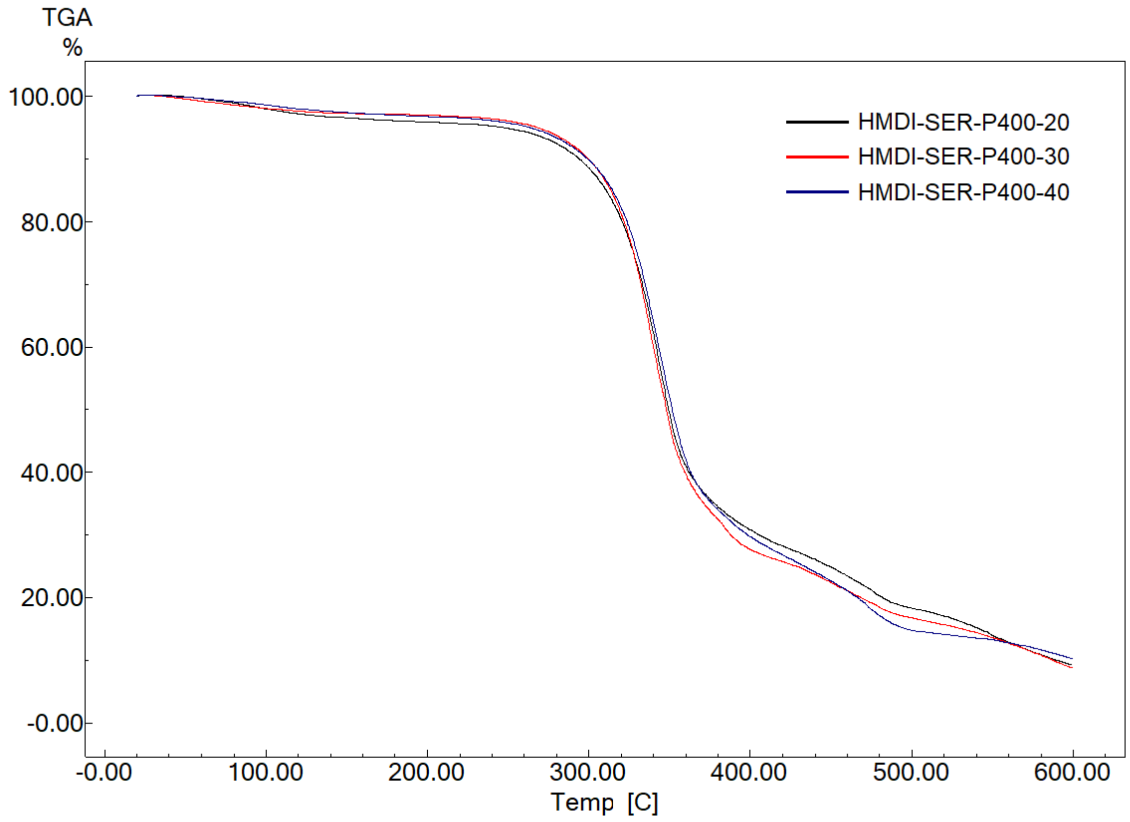

Figure S15. TGA thermograms of HMDI-SER-P400 based polymers

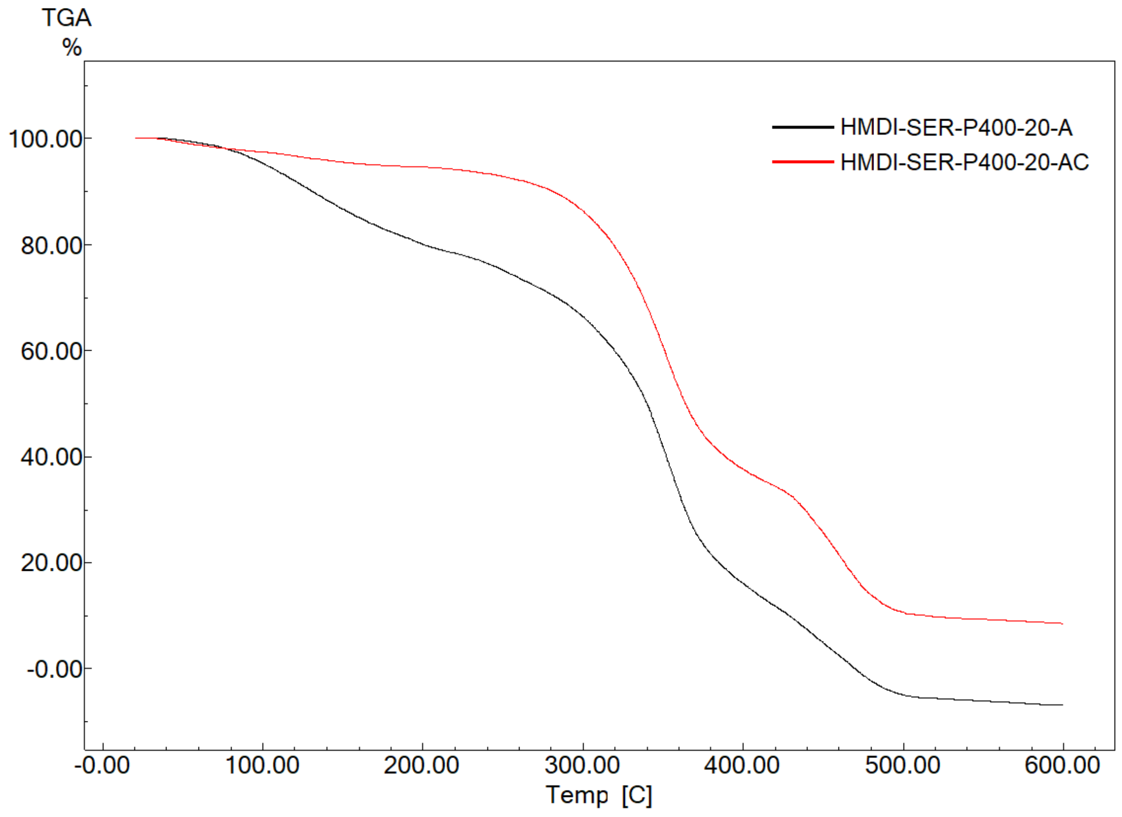

Figure S16. TGA thermograms of HMDI-SER-P400-20 based polymers

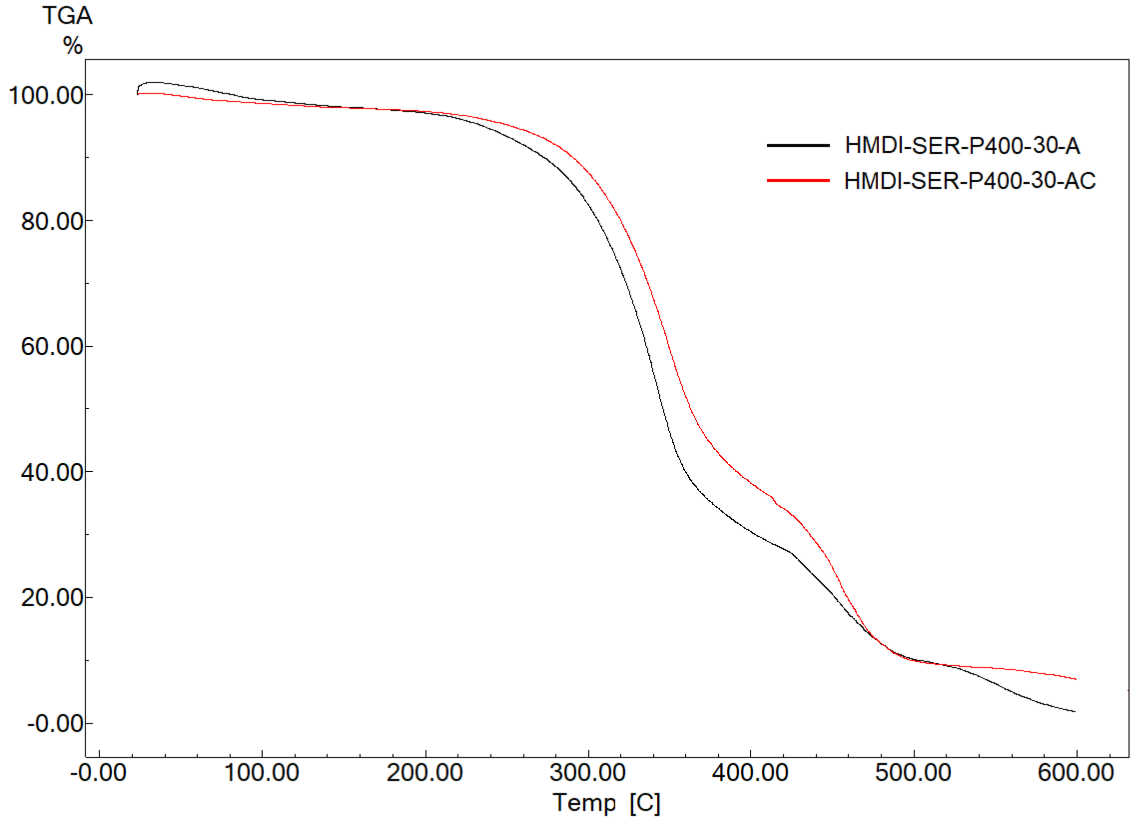

Figure S17. TGA thermograms of HMDI-SER-P400-30 based polymers

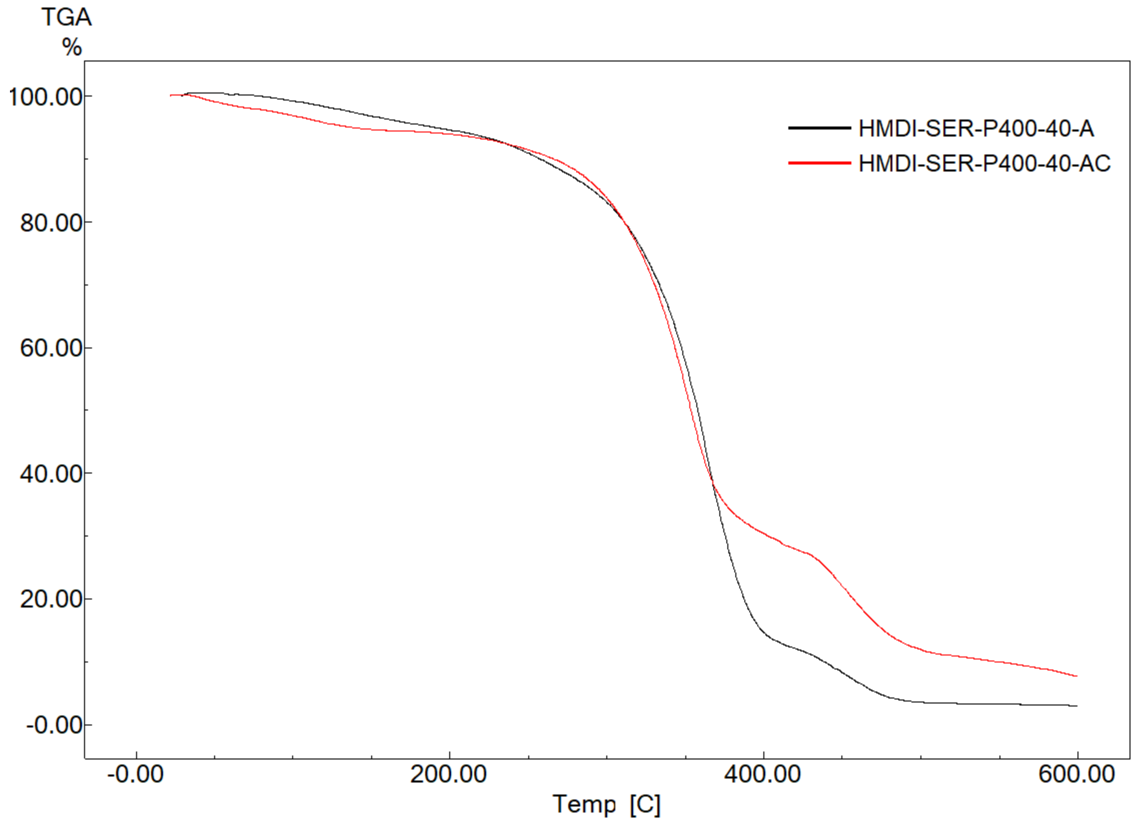

Figure S18. TGA thermograms of HMDI-SER-P400-40 based polymers

FTIR spectra of sericin-based adhesives carrying PEG600 units were given in Figure S19, Figure S20 and Figure S21. In these spectra, the C=O tensile vibration originating from the polyurethane structure was seen at 1708 cm^{-1}. Also, the N-H stress vibration at 3220-3400 cm^{-1} was seen as a medium intensity peak. The N-O peak on the isocyanate bond was observed around 1530 cm^{-1}. These peaks showed that the desired urethane bond was obtained.

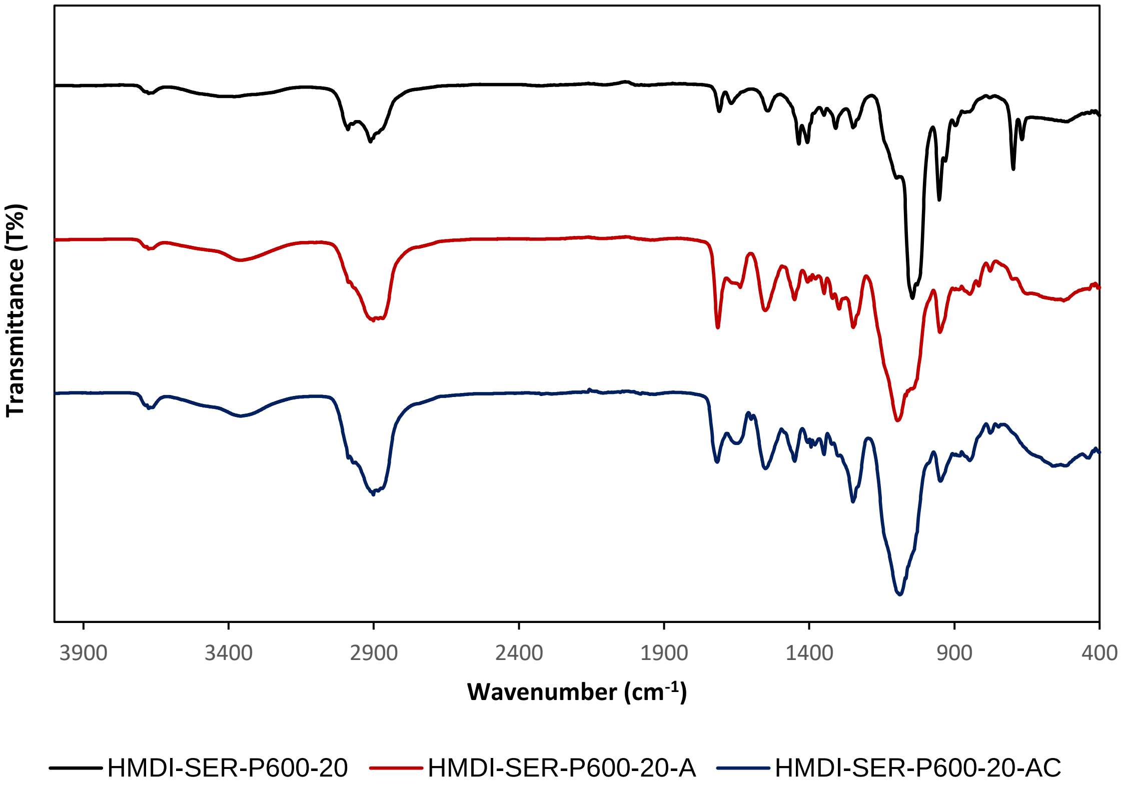

Figure S19. FTIR spectra of HMDI-SER-P600-20 based polymers

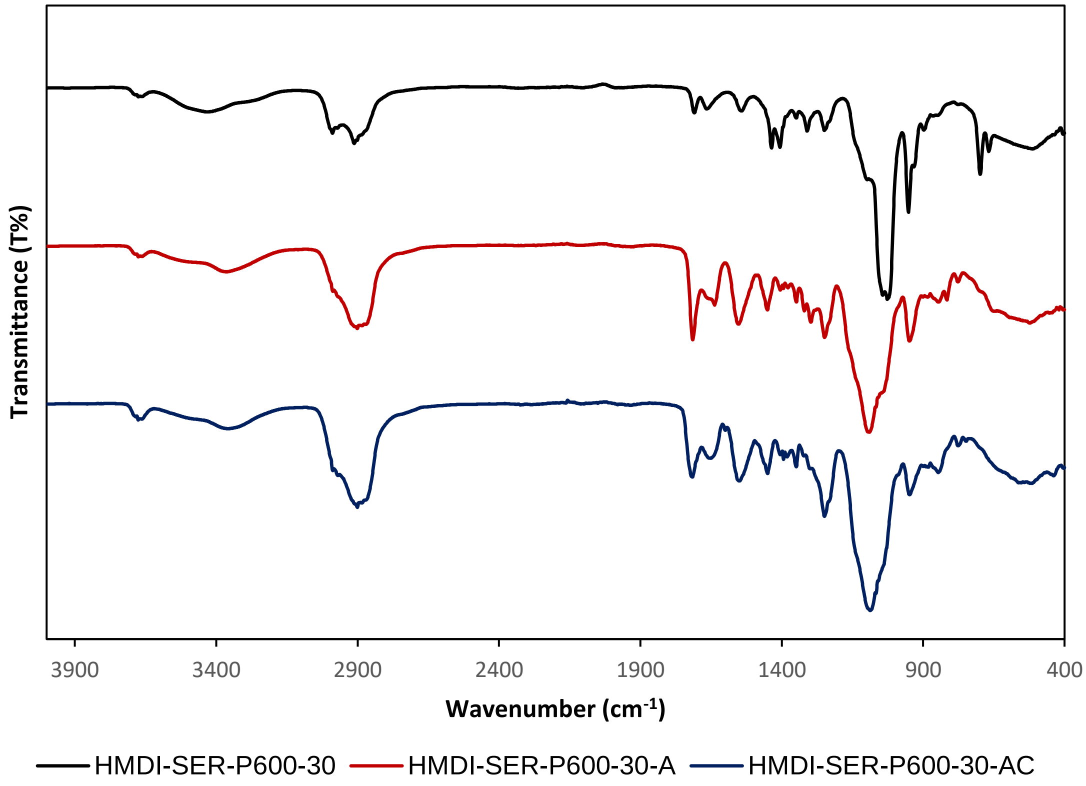

Figure S20. FTIR spectra of HMDI-SER-P600-30 based polymers

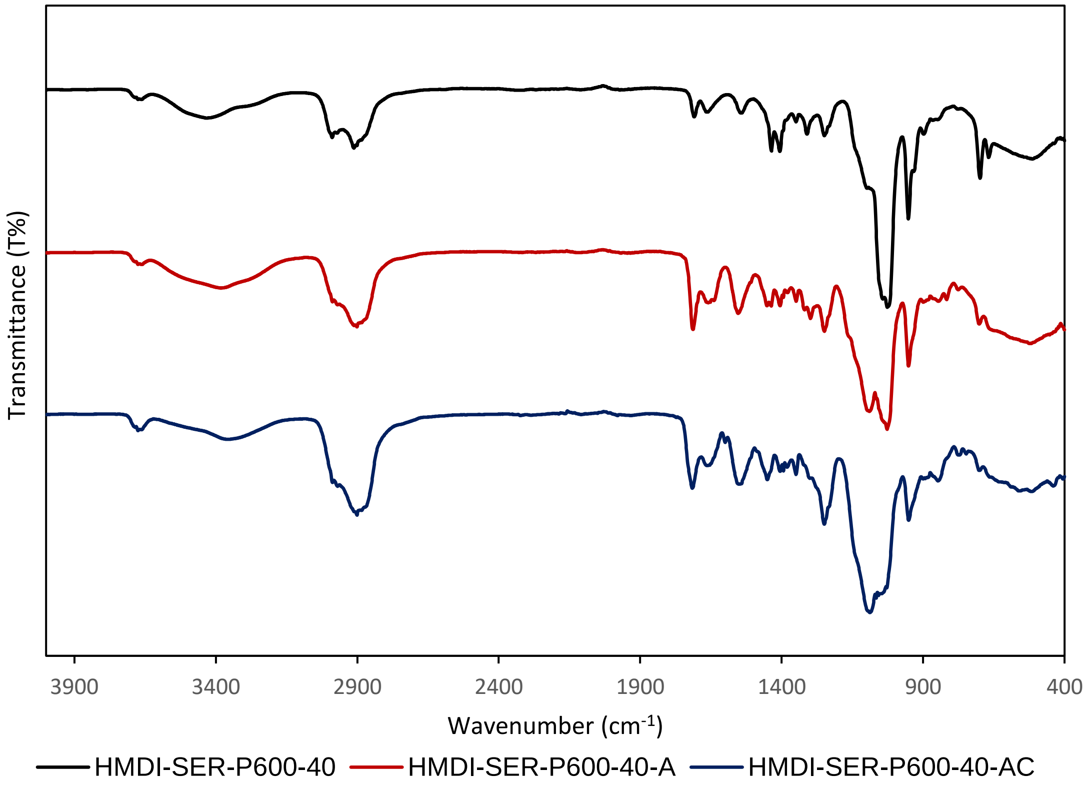

Figure S21. FTIR spectra of HMDI-SER-P600-40 based polymers

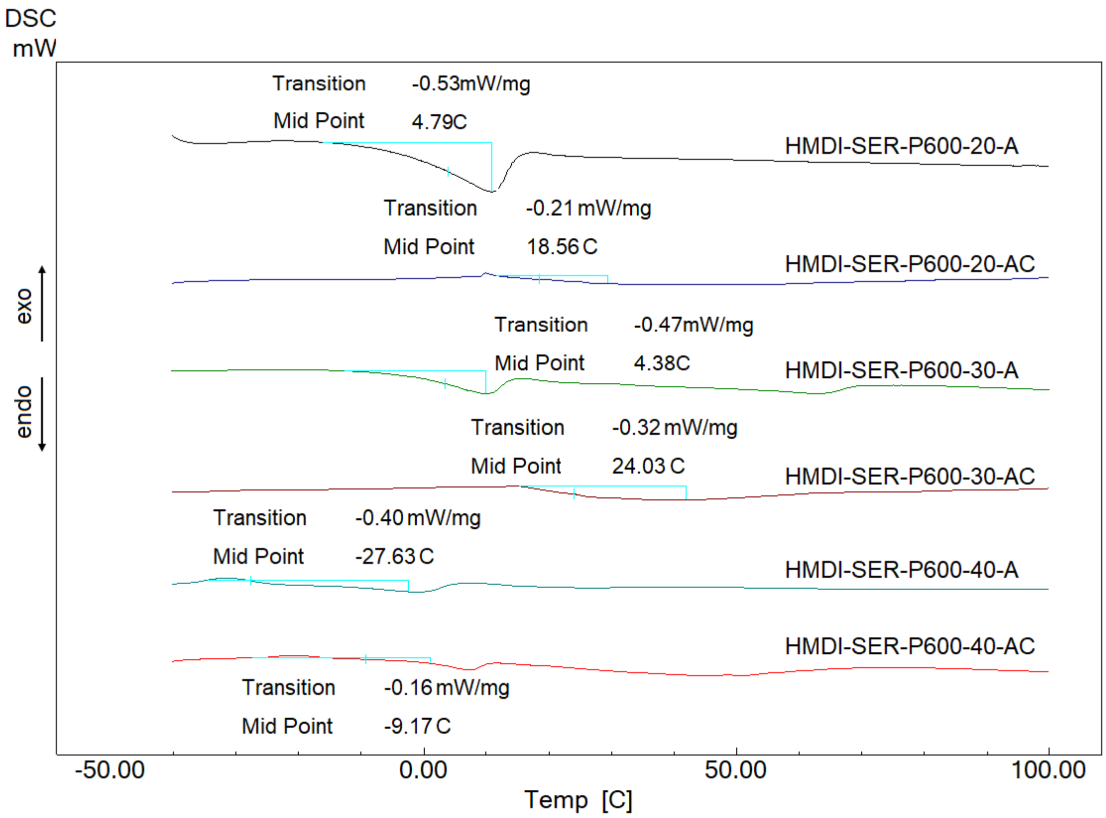

Figure S22. DSC thermograms of HMDI-SER-P600 based polymers

The changes in thermal stability of PEG600-sericin containing polymers were investigated by DTA, TGA and DSC thermograms. These structures were more flexible than other PEG groups. DSC analyzes were performed to determine the Tg values (Figure S22). With the incorporation of acrylic groups in the structure, the Tg values of the polymers were measured as 4.79, 4.38 and -27.63 °C. This value had turned into 18.56, 24.03 and -9.17 °C, respectively, with the UV curing polymerization of acrylic groups. These results showed that when cross-linking formed with curing, the structure becomes harder and the Tg value increases. At the same time, with increasing cross-linking, the free internal volume of the polymer decreases and the thermal stability of the polymeric structure increases. DTA thermograms in Figure S23 support these results. There are three main mass losses of polymers in the TGA thermogram in Figure S24, Figure S25 and Figure S26. The first loss of mass is caused by the removal of the polymer moisture structure at low temperature. The second mass loss is due to the breakage of groups such as CH_2-CH_2-O-CH_2 on the polymer chain. The final mass loss is thermoxidative decomposition. These values are carried to

higher temperatures by curing the acrylate groups. The rate of this increase was seen to be more than other PEG structures.

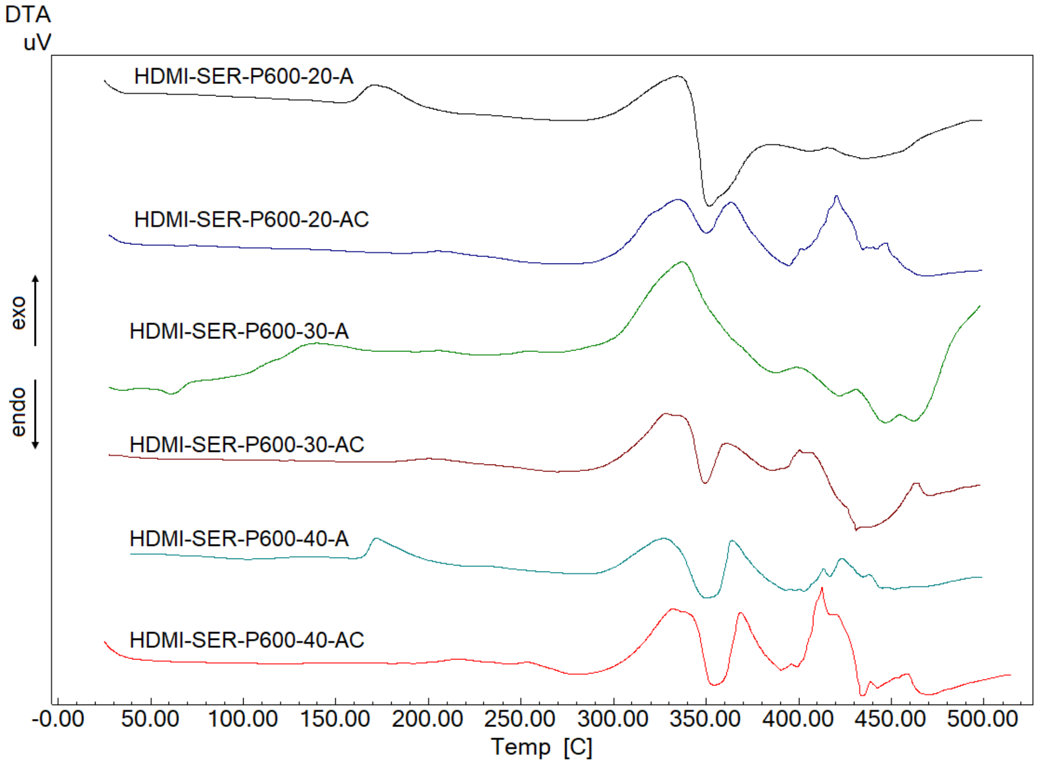

Figure S23. DTA thermograms of HMDI-SER-P600 based polymers

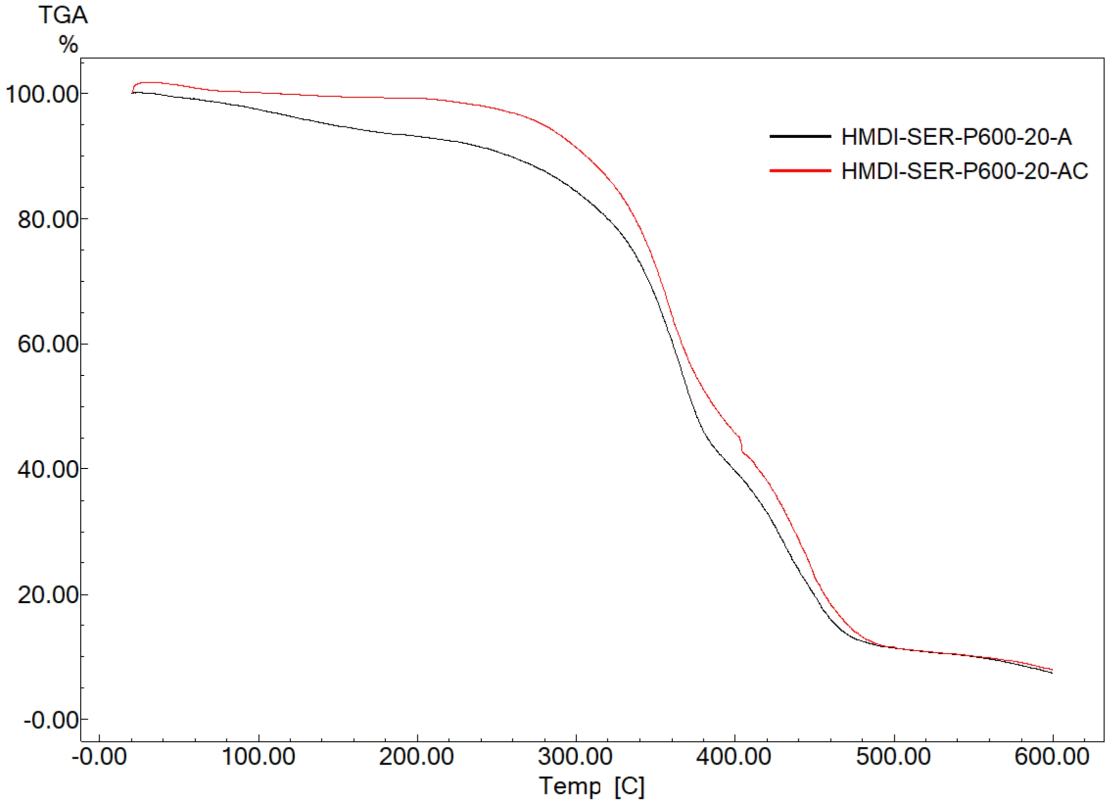

Figure S24. TGA thermograms of HMDI-SER-P600-20 based polymers

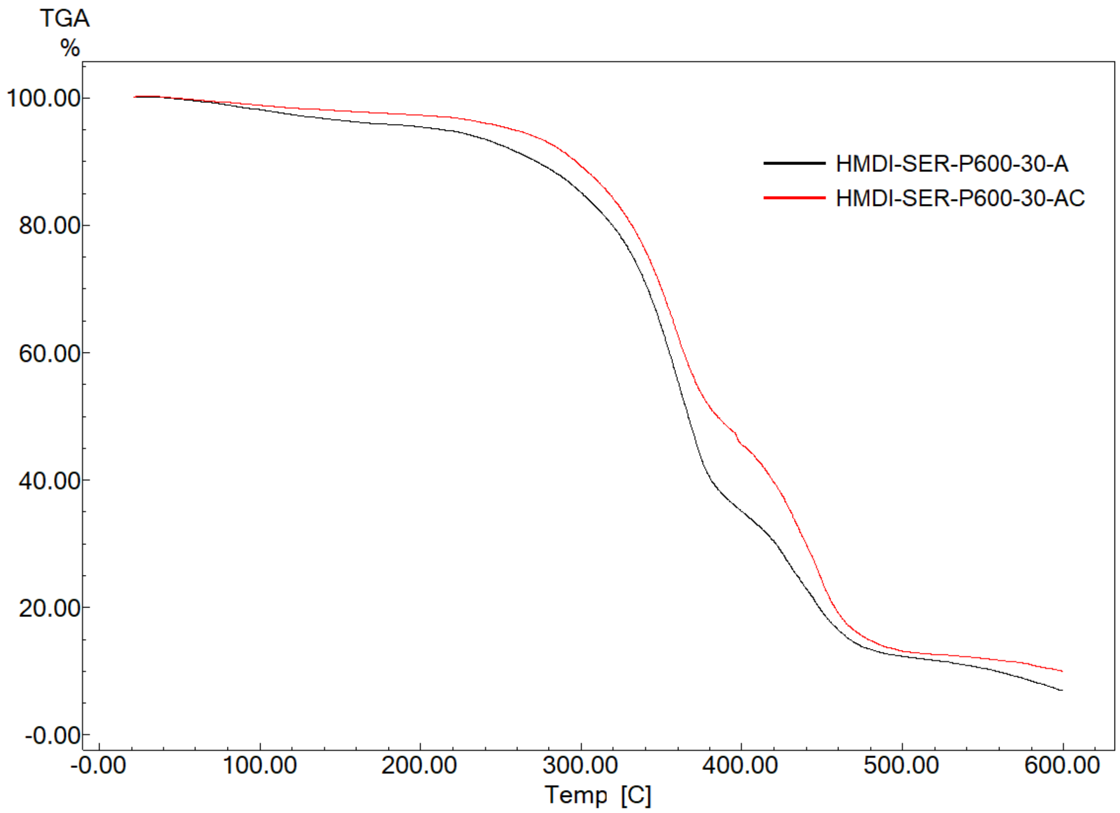

Figure S25. TGA thermograms of HMDI-SER-P600-30 based polymers

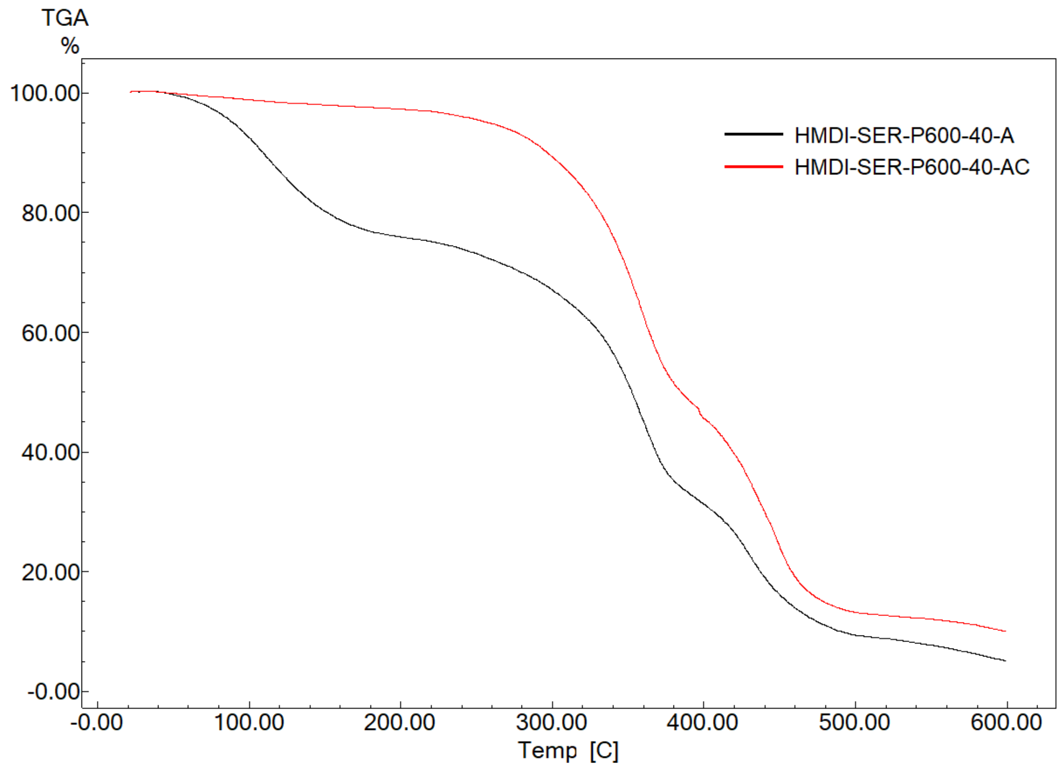

Figure S26. TGA thermograms of HMDI-SER-P600-40 based polymers

NMR Analysis

Acrylate groups were included by linking 2-isocyanatoethyl methacrylate to the synthesized polymers. These structures were determined by NMR spectra to which acrylate groups were attached and interpreted in detail.

NMR spectrum analyzes of HMDI-SER-P400 based adhesive and acrylate bonded structures are given in Figure S26. Primarily, protons originating from aliphatic –CH and –CH_2 groups on the urethane structure show a peak group between 0.7 and 1.9 ppm, respectively. Especially the aliphatic -CH protons originating from the HMDI structure were determined as 3.48(a), 3.42(b) ppm and 1.42(d), 1.39(e) ppm. -CH_2- protons in cyclohexyl groups are seen as 1.49(c) ppm. The peaks of CH_2 protons originating from the PEG units are at 4.05(g), 3.51(h), 3.22(i) and 3.38(i) ppm. The –NH peaks prove the polyurethane structure at 7.23(f). Aliphatic protons originating from cyclodextrin units are seen as peaks around 1.81, 1.72, 1.13, 0.82 and 4.6 ppm. Sericin group peaks are p2, p3 peaks and originate from aromatic amino acids. Based on all these findings, the NMR spectrum in Figure S27 proves that the desired polyurethane structure has been achieved. Acrylate groups are bonded to polyurethane in order to provide UV-curable property. Acrylate groups were examined by NMR spectrum. In Figure S27B, 3 protons with different chemical environment are seen on acrylate groups. These protons are seen at 5.61 ppm and 6.24 ppm(a1), 6.18(a2) ppm and 1.85(a3) ppm. Especially the a3 peak, which has a sharp singlet appearance, is due to the –NH proton in acrylate structure and proves that the acrylate group is included in the structure. The hard and solid polymeric structure formed after curing is another proof of this. In the NMR spectrum in Figure S27B, the polyurethane structure peaks are given. These are seen in the spectrum as g, h, i and j peaks from PEG, 1-9 peaks from cyclodextrin, a, b, c, d, e peaks from isocyanate and p1, p2, p3, p4 peaks from sericin group in the structure. In addition, f peak at 7.20 ppm proved that the urethane bond was formed. As a result, both the pre-polymer structure and the acrylate-bonded polyurethane structure are confirmed in Figure S27.

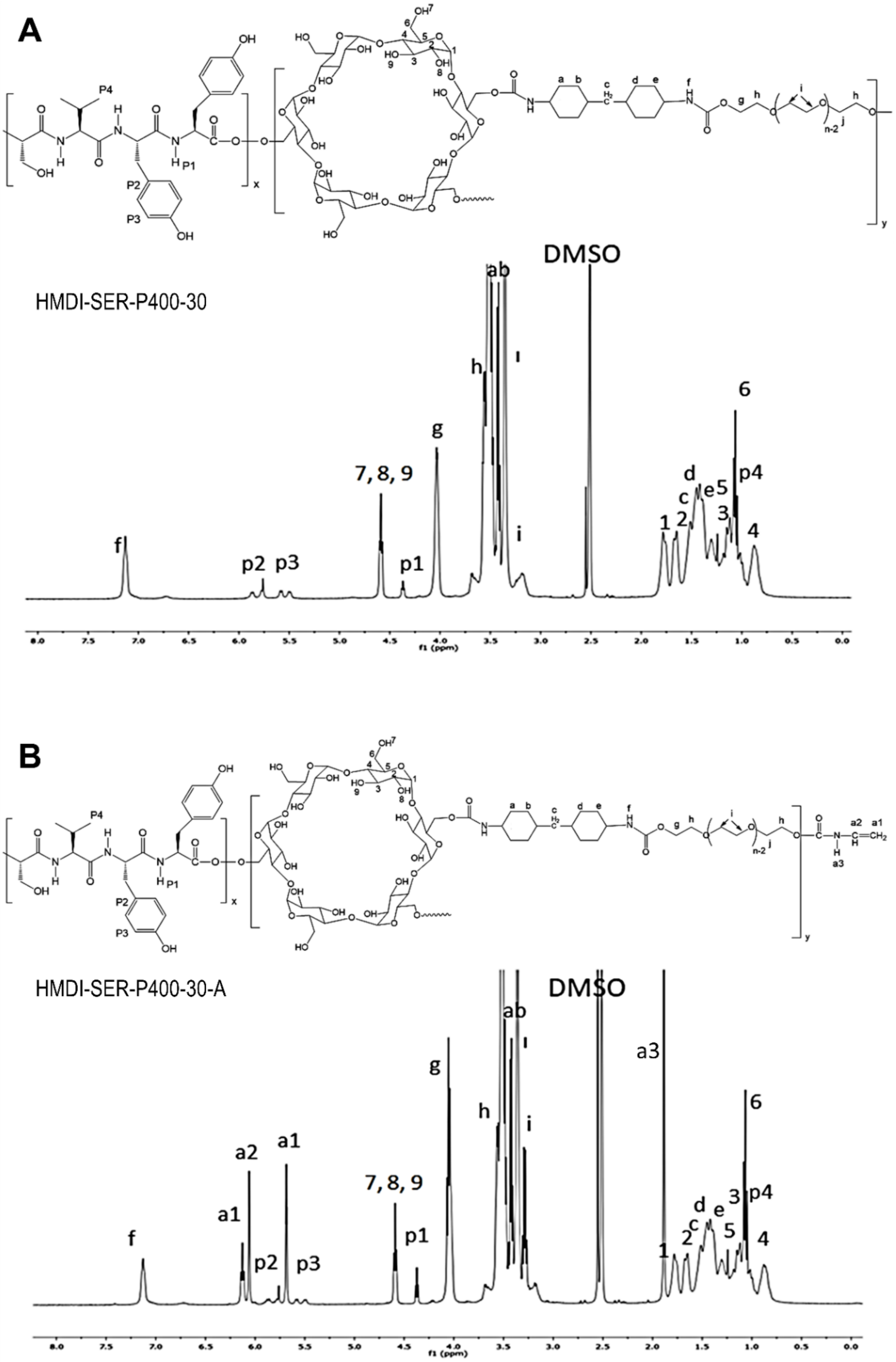

Figure S27. ^{1}H-NMR spectra of HMDI-SER-P400-30 and HMDI-SER-P400-30-A based polymers

Measurement of Free Hydroxyl Number of Polyurethanes

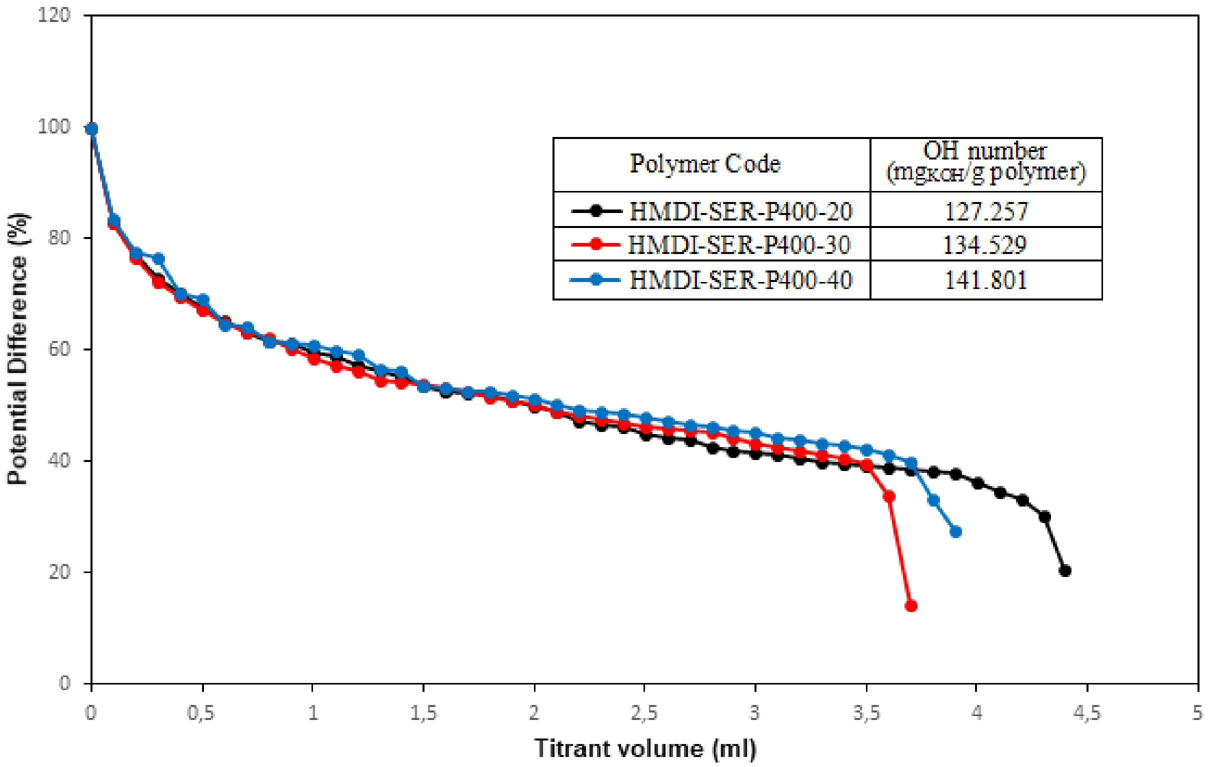

Figure S28. Potentiometric titration curve and OH numbers for HMDI-SER-P400 formulations

SEM and AFM Analysis

Within the study, SEM analyzes were carried out to determine the morphological properties of PU-A formulations (Fig. S29). In these analyzes, UV curing was performed after applying the polymer solution to a glass surface. The cured polymer was removed from the surfaces and SEM analyzes were carried out with these films. SEM images of flat and fractal surfaces from polymers were obtained 1000x and 5000x magnification (Fig. S29). According to the results, polyurethane formed a homogeneous film after curing (PU-A). The detailed analysis of polymer surfaces was also examined with AFM technique (Fig. S30). According to the results, it is seen that the surface roughness is below 50 nm.

Therefore, the polymer structure spreads very well on the applied surfaces and creates a very thin film.

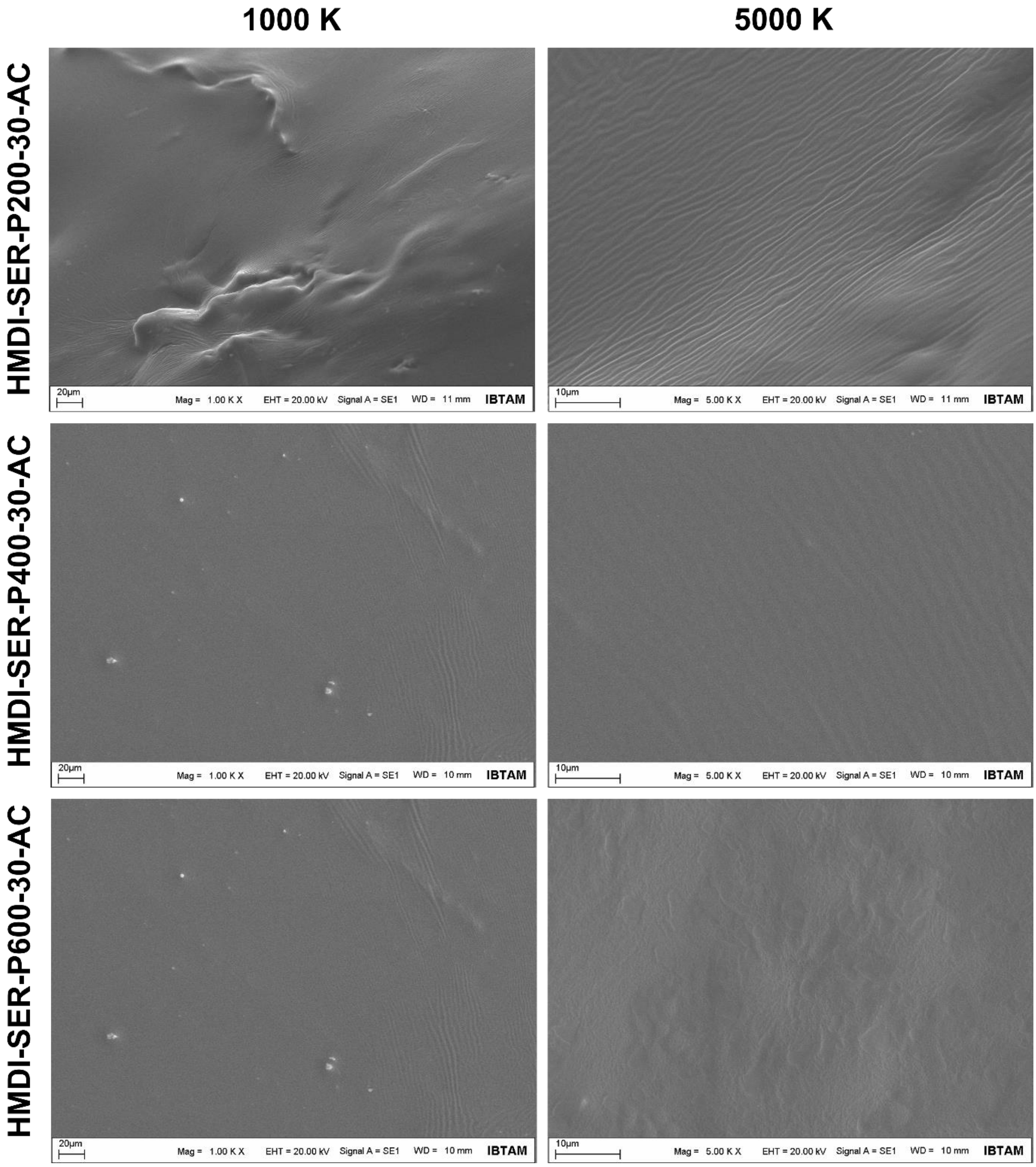

Figure S29. Film surfaces of HMDI-SER-P200, HMDI-SER-P400 and HMDI-SER-P600 by SEM analysis

HMDI-SER-P400-30-AC

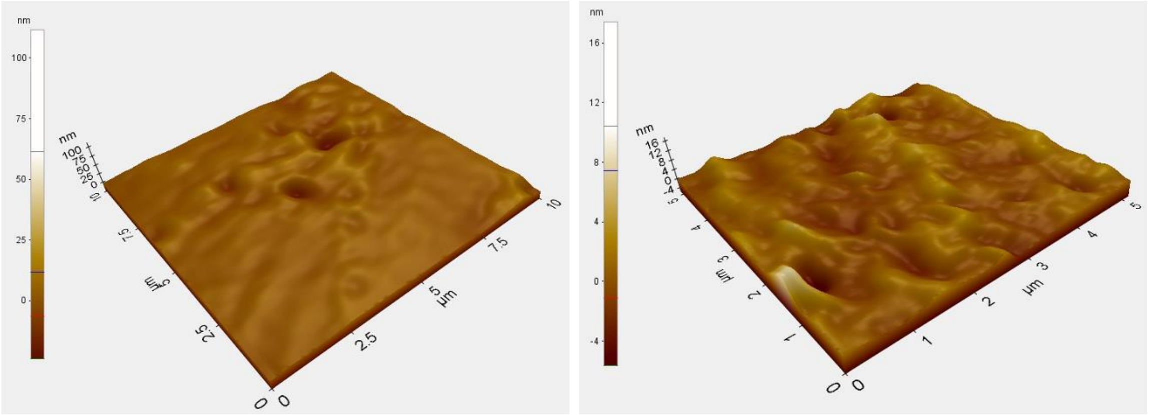

Figure S30. AFM images of the surface of HMDI-SER-P400

Table S1. Adhesion test results of the formulations (kPa)

Sample Code	NCO:OH		
	1:1.2	1:1.3	1:1.4
HMDI-SER-P200	4322.1±214.9	4108.7±72.2	3607.5±60.4
HMDI-SER-P400	3744.5±344.2	3346.8±120.4	2393.6±126.7
HMDI-SER-P600	2558.4±8.4	2667.0±48.8	2071.9±60.2

A

**Groups**	**Inflammation**
Control	0.0 (0.0-0.0)
Steel wire	1.0 (0.0-3.0)[a, b]
Cyanoacrylate	1.0 (0.0-3.0)[a, b]
HMDI-SER-P200-20-AC	1.0 (0.0-3.0)[a]

B

Groups	Inflammation
Control - Steel wire	<0.001
Control - Cyanoacrylate	<0.001
Control -HMDI-SER-P200-20-AC	<0.001
Steel wire - Cyanoacrylate	0.208
Steel wire -HMDI-SER-P200-20-AC	0.009
Cyanoacrylate-HMDI-SER-P200-20-AC	<0.001

Figure S31. A) Inflammation scores of all groups in sternal closure model; B) p values.

[a] Significantly higher compared to the control group ($p<0.05$).

[b] Significantly higher compared to HMDI-SER-P200-20-AC (adhesive formulation) group ($p<0.05$).

References

(1) Chai, K. L.; Noor, I. M.; Aung, M. M.; Abdullah, L. C.; Kufian, M. Z. Non-Edible Oil Based Polyurethane Acrylate with Tetrabutylammonium Iodide Gel Polymer Electrolytes for Dye-Sensitized Solar Cells. *Sol. Energy* **2020**, *208*, 457–468. https://doi.org/10.1016/j.solener.2020.08.020.

(2) Baysal, G.; Keleş, G.; Kalav, B.; Güner, F. S.; Karagüzel Kayaoğlu, B. Synthesis of Ultraviolet (UV)-Curable Water-Borne Polyurethane Acrylate Binders and Comparison of Their Performance for Pigment Printing on Synthetic Leather. *Int. J. Cloth. Sci. Technol.* **2020**, 1–19. https://doi.org/10.1108/IJCST-02-2020-0026.

(3) Mudri, N. H.; Abdullah, L. C.; Aung, M. M.; Salleh, M. Z.; Biak, D. R. A.; Rayung, M. Comparative Study of Aromatic and Cycloaliphatic Isocyanate Effects on Physico-Chemical Properties of Bio-Based Polyurethane Acrylate Coatings. *Polymers (Basel).* **2020**, *12* (7), 1–17. https://doi.org/10.3390/polym12071494.

(4) Sultan, M.; Bhatti, H. N.; Zuber, M.; Barikani, M. Synthesis and Characterization of Waterborne Polyurethane Acrylate Copolymers. *Korean J. Chem. Eng.* **2013**, *30* (2), 488–493. https://doi.org/10.1007/s11814-012-0148-3.
